# Early stage β-amyloid-membrane interactions modulate lipid dynamics and influence structural interfaces and fibrillation

**DOI:** 10.1016/j.jbc.2022.102491

**Published:** 2022-09-14

**Authors:** June M. Kenyaga, Qinghui Cheng, Wei Qiang

**Affiliations:** Department of Chemistry, Binghamton University, The State University of New York, Vestal, New York, USA

**Keywords:** amyloid-beta, membrane, solid-state NMR, biophysics, Alzheimer disease, Aβ, β-amyloid, BCA, bicinchoninic acid, CP, crosspolarization, DMSO, dimethyl sulfoxide, MD, molecular dynamics, MPL, mass per length, N2a, Neuro-2a, RDC, residual dipolar coupling, REDOR, rotational-echo double-resonance, ssNMR, solid-state NMR, TEM, transmission electron microscopy, ThT, thioflavin-T, TMV, tobacco mosaic virus

## Abstract

Molecular interactions between β-amyloid (Aβ) peptide and membranes contribute to the neuronal toxicity of Aβ and the pathology of Alzheimer’s disease. Neuronal plasma membranes serve as biologically relevant environments for the Aβ aggregation process as well as affect the structural polymorphisms of Aβ aggregates. However, the nature of these interactions is unknown. Here, we utilized solid-state NMR spectroscopy to explore the site-specific interactions between Aβ peptides and lipids in synaptic plasma membranes at the membrane-associated nucleation stage. The key results show that different segments in the hydrophobic sequence of Aβ initiate membrane binding and interstrand assembling. We demonstrate early stage Aβ-lipid interactions modulate lipid dynamics, leading to more rapid lipid headgroup motion and reduced lateral diffusive motion. These early events influence the structural polymorphisms of yielded membrane-associated Aβ fibrils with distinct C-terminal quaternary interface structure compared to fibrils grown in aqueous solutions. Based on our results, we propose a schematic mechanism by which Aβ-lipid interactions drive membrane-associated nucleation processes, providing molecular insights into the early events of fibrillation in biological environments.

Neuronal plasma membrane is highly biologically relevant to the pathological aggregation of β-amyloid (Aβ) peptides. Enzymatic cleavage of amyloid precursor protein by γ-secretase occurs in membrane interior to produce Aβ sequences (*i.e.*, 37–43 residues), which consequently release to the exocellular environments (1, 2). Senile amyloid plaques from Alzheimer’s patients contain extracellular matrix and cell surface proteins as well as membrane compositions such as cholesterol, sphingolipids, and gangliosides (3, 4). Direct interactions between Aβ peptides and membrane components contribute to both the pathological and physiological roles of Aβ. Different membrane disruptive effects, including pore/ion channel formation (5, 6, 7), membrane fragmentation (8), lipid uptake (9), content leakage (10, 11, 12), and vesicle fusion (13), have been identified under various experimental conditions using model phospholipid-based liposomes. In living cells, incorporation of Aβ oligomers has been shown to induce pore formation (14) and modulations of neuronal membrane elasticity (15, 16). Physiologically, recent studies suggest that Aβ may act as antimicrobial peptides in innate immune system because it may form membrane pores to kill bacteria and fungi (17, 18).

Despite its high biological relevance, fundamental questions about Aβ-membrane interactions and its pathologically relevant consequences remain unaddressed. For instance, kinetics studies have suggested that interactions between Aβ and membrane components at nucleation stage disrupted membrane bilayers, as membrane content leakage occurred prior to the fibrillation of Aβ in model bilayers (10, 11, 19, 20, 21). Therefore, exploration of early stage Aβ-membrane interactions would provide insights on the mechanism of Aβ cytotoxicity. However, very few high resolution studies target these early stage events. In addition, the presence of membrane is known to modulate the aggregation kinetics (20, 22, 23, 24, 25) and the local conformation and higher order architecture of Aβ aggregates (26, 27, 28, 29). However, high resolution structural data that unravel the impact of membrane on the structural polymorphisms of resulted fibrils are rare. Particularly, the structural polymorphism of Aβ fibrils could be pathologically relevant because recent studies on Aβ fibrils from Alzheimer’s patients’ brain tissue suggested its potential correlation with the patients’ clinical symptoms (30, 31, 32, 33). The answers to the aforementioned two unaddressed questions may further be correlated. Previous works suggested that Aβ underwent distinct fibrillation pathways in the presence of membrane comparing with the scenario in aqueous solutions (8, 20, 34, 35). It is likely that the early stage structural changes of Aβ that produce fibrillar nuclei are accompanied by Aβ-membrane interactions, and structural features in these membrane-associated nuclei may propagate to mature fibrils.

To shed light on these questions, we explore the early stage Aβ-lipid interaction within the nucleation period of membrane-associated fibrillation process of 40-residue Aβ (Aβ_1–40_) using mainly solid-state NMR (ssNMR) spectroscopy. Synaptic plasma membranes extracted from rats’ brain tissues (rSPMs) serve as a biologically relevant membrane model in the present work. Site-specific Aβ-Aβ and Aβ-lipid proximities and their time-dependent changes were assessed. Lipid dynamics within the same time course were monitored to elucidate the modulations of membrane properties induced by Aβ-lipid interactions. The molecular structure of mature rSPMs-Aβ_1−40_ fibril was determined, where distinct structural features were identified within the membrane-interacting core segment.

## Results

### Aβ_1–40_ adopts a membrane-associated nucleation process in the presence of rSPMs

**Figure 1:**
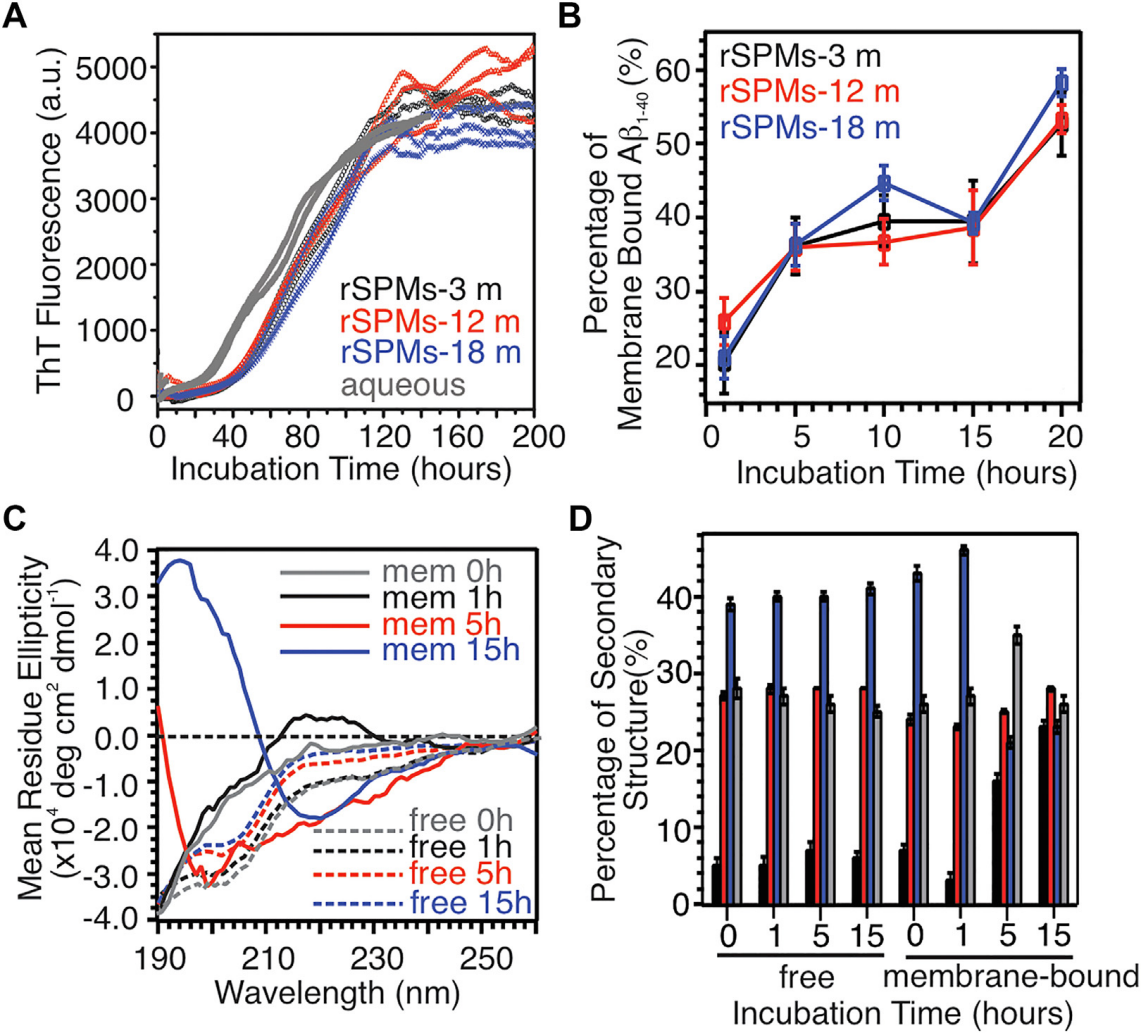
**Biophysical characterization of rSPMs-bound Aβ**_**1–40**_**.***A*, ThT fluorescence kinetics of Aβ_1–40_ fibrillation in rSPMs with different ages and in 10 mM phosphate buffer (2–3 representative repetitions for each condition) The best-fit lag periods for fibrillation are: 39.4 ± 1.8 h for 3 month rSPMs; 37.2 ± 1.8 h for 12 month rSPMs; 40.5 ± 1.7 h for 18 month rSPMs; and 28.5 ± 1.4 h for aqueous buffer. *B*, binding of Aβ_1–40_ to rSPMs with different ages, studied by BCA assay. *C*, representative CD spectra of membrane-bound (*solid lines*) and free (*dashed lines*) Aβ_1–40_ with different incubation time periods from 0 to 15 h. *D*, plots of secondary structure populations extracted by analyzing the CD spectra using CDPro software. The uncertainties were determined from three repetitions for each sample conditions. Aβ, β-amyloid; ThT, thioflavin-T.

Figure 1*A* shows the thioflavin-T (ThT) fluorescence kinetics of fibrillation of 10 μM Aβ_1–40_ in the presence of rSPMs extracted from 3 to 18 month rats brain tissues, with 37 to 40 h lag periods. Compared with the lag period of Aβ_1–40_ fibrillation in aqueous solution (*e.g.*, ∼28 h), the presence of rSPMs decelerates the nucleation process. The aqueous lag period values obtained in the current work agree in general with two previous comparable studies, where the ThT-based lag periods of Aβ_1–40_ fibrillation was ∼25 h at 10 μM (36) and 5 to 15 h at 50 μM (37). Quantification of the membrane-bound Aβ_1–40_ within the lag period using bicinchoninic acid (BCA) assay (Fig. 1*B*) shows an increasing trend with about 20% to 25% instant binding, which is consistent with previous characterizations in phospholipid vesicles (20). Time evolution of the globular secondary structures of free and membrane-bound Aβ_1–40_ in 12 month rSPMs was monitored using CD spectroscopy (Fig. 1, *C* and *D*). Membrane-bound Aβ_1–40_ shows transition from predominant random coil to more ordered conformations within 15 h, while the structure of free Aβ_1–40_ peptides remain unchanged, suggesting a membrane-associated nucleation process. Further evidence from FTIR spectroscopy shows that the parallel-β strand conformation in membrane-bound Aβ_1–40_ but not in free peptides increases within 15 h incubation time (Fig. S1).

### Early stage site-specific interactions between Aβ_1−40_ and lipids reveal distinct membrane-binding and nucleation segments

We performed ssNMR spectroscopy on single-site isotope labeled Aβ_1–40_ samples to probe the early stage molecular interactions. The peptides were incubated with rSPMs for 5 and 15 h under physiological conditions, within the ThT fluorescence–based lag periods. ^13^C-^31^P rotational-echo double-resonance (REDOR) (38) and ^13^C-PITHIRDs-CT (39) spectroscopy were used to map the internuclear distances between selected sites and lipid phosphate headgroups and between adjacent peptide strands, respectively. In the present work, we select seven residues, F19, A21, G25, G29, A30, L34, and V36, located in the core segments of reported Aβ_1–40_ fibrils (32, 40, 41, 42).

**Figure 2:**
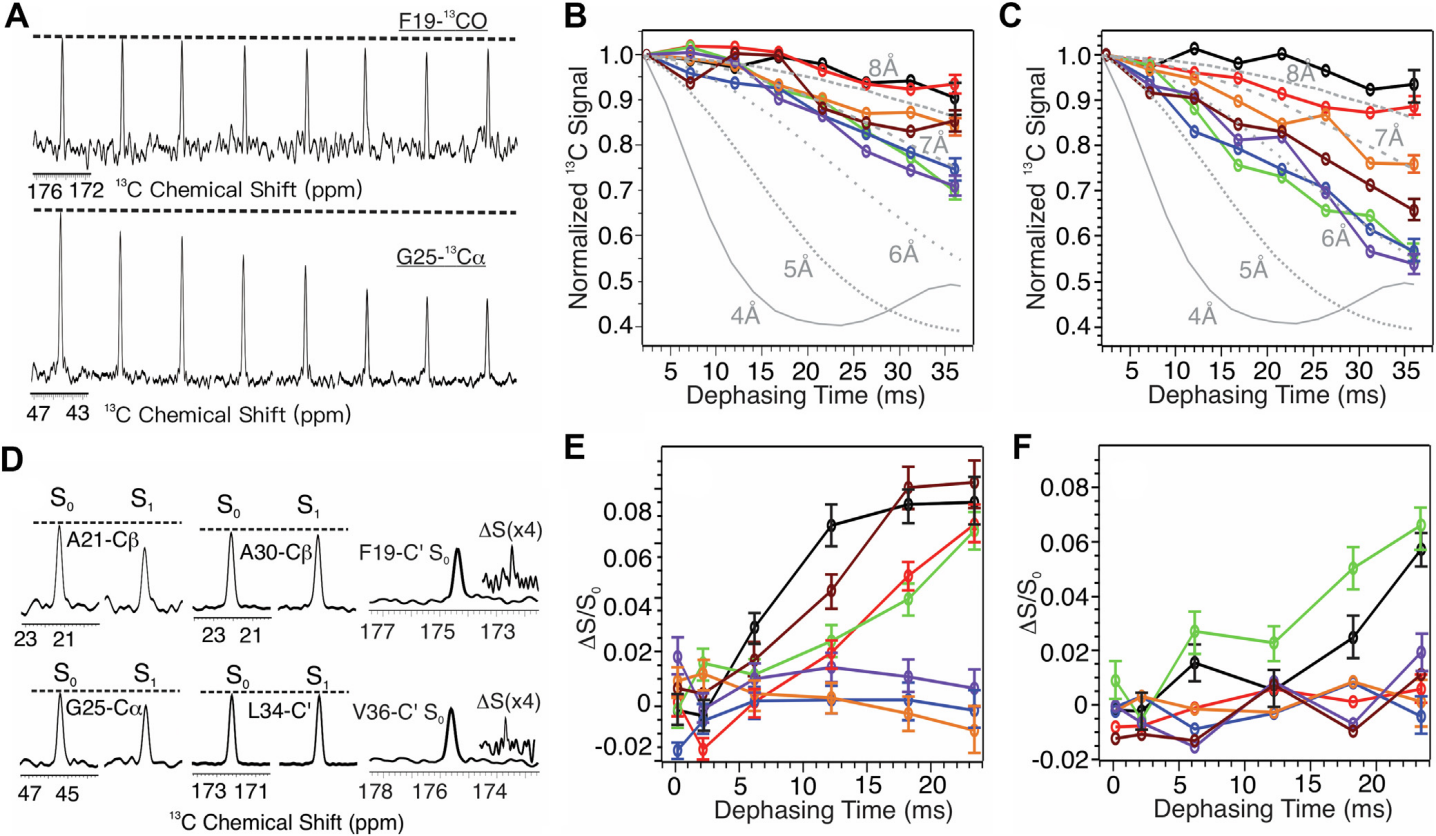
^**13**^**C-PITHIRDs-CT and**^**13**^**C-**^**31**^**P REDOR spectra and dephasing curves.***A*, representative ^13^C-PITHIRDs-CT spectra for two singly isotope-labeled sites. *B* and *C*, experimental decay curves for individual labeled sites with 5 h (panel *B*) and 15 h (panel *C*) incubation. *Gray lines* are simulated ^13^C-PITHIRDs-CT decay curves with ^13^C-^13^C internuclear distances from 4 to 8 Å. *D*, representative REDOR S_0_ (full), S_1_ (reduced), and ΔS (difference) spectra for individual isotope-labeled sites, collected with 24 ms dephasing time for samples with 5 h incubation. *E* and *F*, REDOR build-up curves for individual labeled sites with 5 h (panel *E*) and 15 h (panel *F*) incubation. Color-coding in (*B*), (*C*), (*E*), and (*F*) for individual sites: *black*, F19-C’; *red*, A21-Cβ; *green*, G25-Cα; *blue*, G29-Cα; *orange*, A30-Cβ; *purple*, L34-C’; *brown*, V36-C’. REDOR, rotational-echo double-resonance.

Figure 2*A* shows representative ^13^C-PITHIRDs-CT spectra at two different sites, F19-C′ and G25-Cα. Additional spectra that lead to the decay curves plotted in Figure 2*B* are provided in Fig. S2. Faster decay of ^13^C peak intensities indicates stronger ^13^C-^13^C dipolar coupling and, therefore, shorter interstrand distance. Interstrand distances at all labeled sites become shorter from 5 to 15 h (*e.g.*, from ∼7 to ∼6 Å at G25, G29, and L34), indicating assembling of peptides and formation of oligomers within nucleation period. None of these residues shows ∼5 Å interstrand distance, indicating that the early stage rSPMs-Aβ_1–40_ oligomers do not possess parallel-in-register β sheet structures as in mature fibrils. At residue-specific level, G25, G29, A30, L34, and V36 show closer interstrand contact and therefore earlier time-dependent chain assembling, while sites F19 and A21 are farther away, indicating that the C-terminal segment of Aβ_1–40_ sequence assembles first in the rSPMs-associated nucleation process. Interestingly, early stage CD and FTIR spectra showed the formation of β sheets at 15 h incubation time. However, the ThT-fluorescence kinetics measurements on rSPMs-associated Aβ_1–40_ fibrillation revealed 30 to 40 h lag periods. Insights from residue-specific PITHIRDs-CT data provide a possible explanation: At 15 h, none of the selected residues in the fibrillar core form parallel-in-register β sheets, confirmed by 6∼8 Å interstrand distances. Therefore, although certain β sheet structures may form rapidly (*e.g.*, 15 h), parallel-in-register β sheets structural motifs may be required for either effective elongation and/or fluorescent-active ThT binding.

Next, we probe the early stage site-specific Aβ_1–40_-lipid interactions within the same period. Figure 2*D* shows representative ^13^C-^31^P REDOR spectra and Figure 2, *E* and *F* summarize the build-up curves (additional REDOR spectra in Fig. S3). For all samples, only <10% REDOR dephasing (*i.e.*, ΔS/S_0_ < 0.1) were observed, indicating that a large population of Aβ_1–40_ molecules were not in proximity with lipids. The magnitude of REDOR dephasing decreases from 5 to 15 h, opposite to the trend of PITHIRDs-based Aβ_1–40_/Aβ_1–40_ interactions. This is consistent with the formation of oligomers where individual Aβ_1–40_ chains may assemble and therefore become shielded from lipids. The sites F19, A21, G25, and V36 showed closer contact with ^31^Ps at 5 h, and A21 and G25 remained close in contacts with ^31^Ps at 15 h. Comparison between the PITHIRDs and REDOR data suggests that different Aβ_1–40_ segments may involve in early stage peptide assembling and lipid interactions. Notably, residue-specific lipid proximities found in current study are consistent with a recent all-atom molecular dynamics (MD) simulation of Aβ interaction with neuronal membrane mimics, where the segment F20-A30 was identified as a region that interacted strongly with membrane (43).

### Modulation of lipid dynamics induced by early stage Aβ_1−40_-membrane interactions

We next ask how the early stage Aβ-lipid interactions would affect lipid dynamics, which reports important membrane physicochemical properties such as fluidity and overall curvature. Quantification of lipid dynamics is done by assessing the microsecond (τ_s_) and nanosecond (τ_f_) timescale motion correlation times of lipids using ^31^P ssNMR relaxation spectroscopy (44, 45, 46).

**Figure 3:**
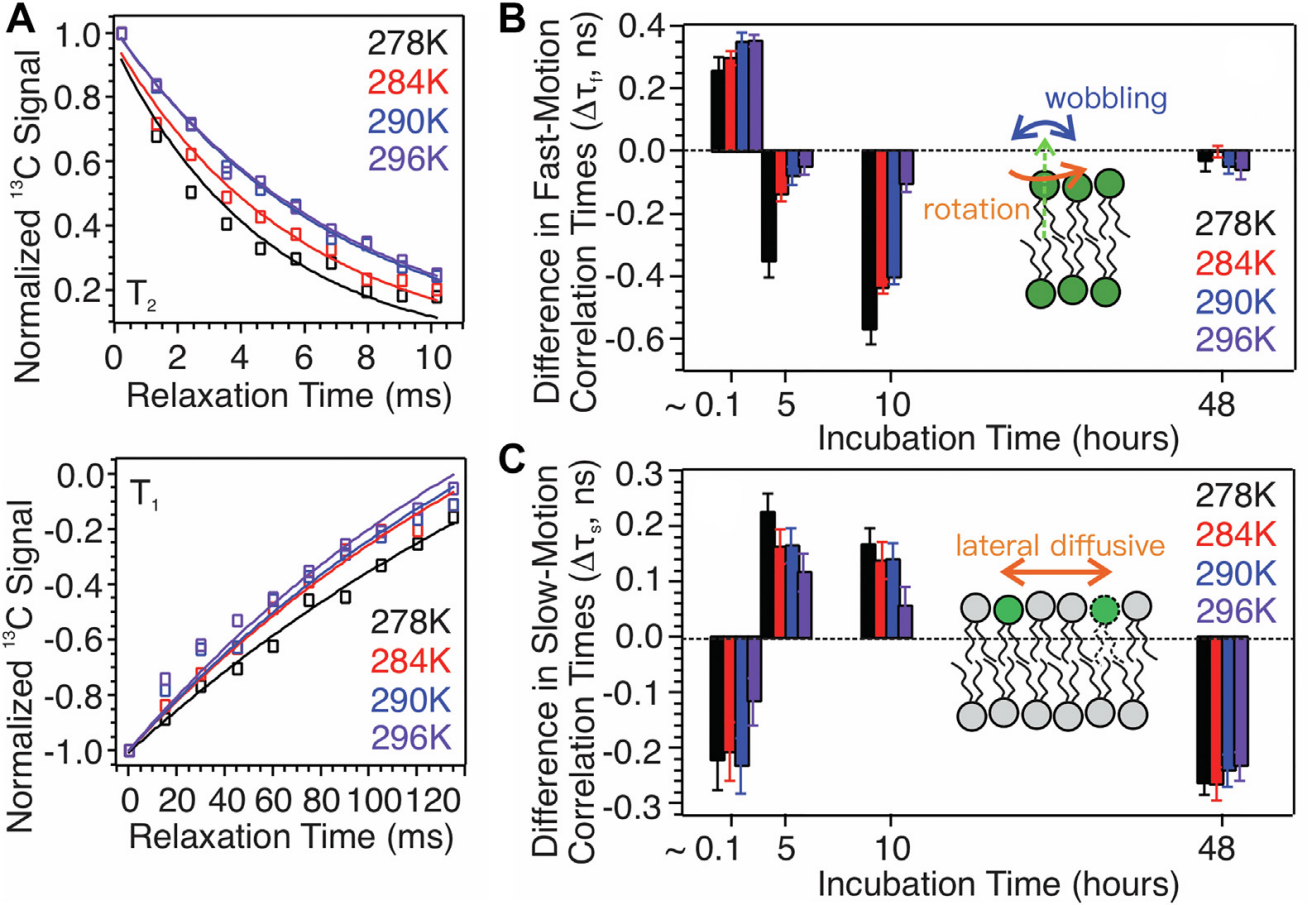
^**31**^**P relaxation measurements on lipid dynamics.***A*, representative ^31^P T2 (*top*) and T1 (*bottom*) relaxation for Aβ_1–40_/membrane samples with 5 h incubation. Experimental data and fitting curves are shown in *open symbols* and *solid lines*, respectively. *B* and *C*, modulations of nanosecond (panel *B*, Δτ_f_) and microsecond (panel *C*, Δτ_s_) timescale correlation times for samples with different incubation times. Positive and negative bars in panel (*B*) and (*C*) indicate the increase and decrease of correlation times due to the addition of Aβ_1–40_ peptides. Predominant lipid motions contributing to the correlation times are shown schematically as *insets*. Uniform color coding for different sample temperatures is provided in panels. Aβ, β-amyloid.

^31^P spin-lattice (T_1_) and spin-spin (T_2_) relaxation curves were recorded at various temperatures from 278 K to 296 K (Figs. 3*A*, S4 and S5). Although splitting of ^31^P peaks were observed, the spectral resolution was insufficient to analyze individual phospholipid types. Therefore, an overall ^31^P intensity was quantified. The values of τ_s_ and τ_f_ were derived from the relaxation time constants (47). In rSPMs, the nanosecond timescale phospholipid headgroup motions such as the uniaxial rotation and wobbling dominate τ_f_ and the main microsecond timescale motion that influences τ_s_ is the lateral diffusive motion (44).

Figure 3, *B* and *C* plot the change of τ_f_ and τ_s_ (*i.e.*, Δτ_f_ and Δτ_s_) in the presence and absence of Aβ_1–40_ peptides at different incubation times. Positive and negative deviation values indicate the restriction and acceleration of corresponding motions due to the addition of Aβ_1–40_ peptides, respectively. Several conclusions can be drawn. First, the incorporation of Aβ_1–40_ peptides with rSPMs induces instant restriction of phospholipid headgroup motions, demonstrated by the noticeable increments of τ_f_ at short incubation time (∼10 min, *x*-axis in Fig. 3*B*). This effect may be attributed to the strong electrostatic interactions between charged residues in Aβ_1–40_ and lipid phosphate groups that drive the initial binding, which were previously shown by MD simulations (48, 49, 50). Second, instant decrease of τ_s_ is observed, suggesting more rapid lipid lateral diffusive motion upon the addition of Aβ. Similar effect has been reported in a previous study using the amyloidogenic Aβ_25–35_ segment and phosphatidylcholine/phosphatidylserine bilayers (51), where the modulation of lipid diffusive motion was attributed to the “fluid mosaic model” where peptides could diffuse freely in a single layer upon initial binding and instantly increased the flexibility of lipids lateral diffusive motion.

Third, modulations of lipid headgroup and diffusive motion correlation times were monitored at 5 and 15 h, matching the time frame of early stage molecular interactions determined by PITHIRDs and REDOR. Changes in τ_f_ and τ_s_ values indicate accelerated lipid headgroup motions and decelerated lateral diffusion. Enhanced lipid headgroup motions may suggest local membrane disruptions, which are consistent with rapid vesicle content leakage within the first 20 h observed in multiple bilayer models (10, 13, 20). Restriction of lipid diffusive motion may be attributed to hydrophobic interactions between Aβ and lipid alkyl chains. MD simulation showed that hydrophobic interactions between Aβ and multiple lipid alkyl chains could drive peptide conformational changes from helices to β-strand at early stages (52). We have also previously shown using ssNMR spectroscopy that lipids could intercalate into the β-sheet hydrogen bonding network of large Aβ oligomers (47). The present PITHIRDs data within the same time frame also suggested nonhelical backbone conformation because several residues possessed ∼6 Å interstrand distances. Such short distances would be unlikely for helical backbone conformation considering the spacing due to side chains but more likely to fit β-strands where the side chains orientate perpendicular to backbones. Therefore, we propose that early stage hydrophobic interactions between low-order Aβ oligomers and lipid alkyl chains in the lag period diminish the lateral diffusive motion of lipids. Finally, modulations of correlation times were recorded at 48 h incubation time, which was within the elongation phase of fibrillation. The presence of Aβ does not show significant influence on lipid headgroup motions but accelerates the diffusive motion. The latter may be attributed to the membrane curvature changes caused by the surface adherence of Aβ fibrils, which have been reported in previous studies (5, 22, 23).

### Distinct structural polymorphisms in rSPMs-Aβ_1–40_ fibrils

The molecular structural polymorphism induced by membrane-associated Aβ fibrillation has not been fully understood. Following our results that Aβ_1−40_-lipid interactions occur within the same time frame as the initial peptide chain assembling, we asked whether such interactions would cause structural polymorphisms in the resultant fibrils and how such structural changes may influence the chemical and biological features of fibrils.

**Figure 4:**
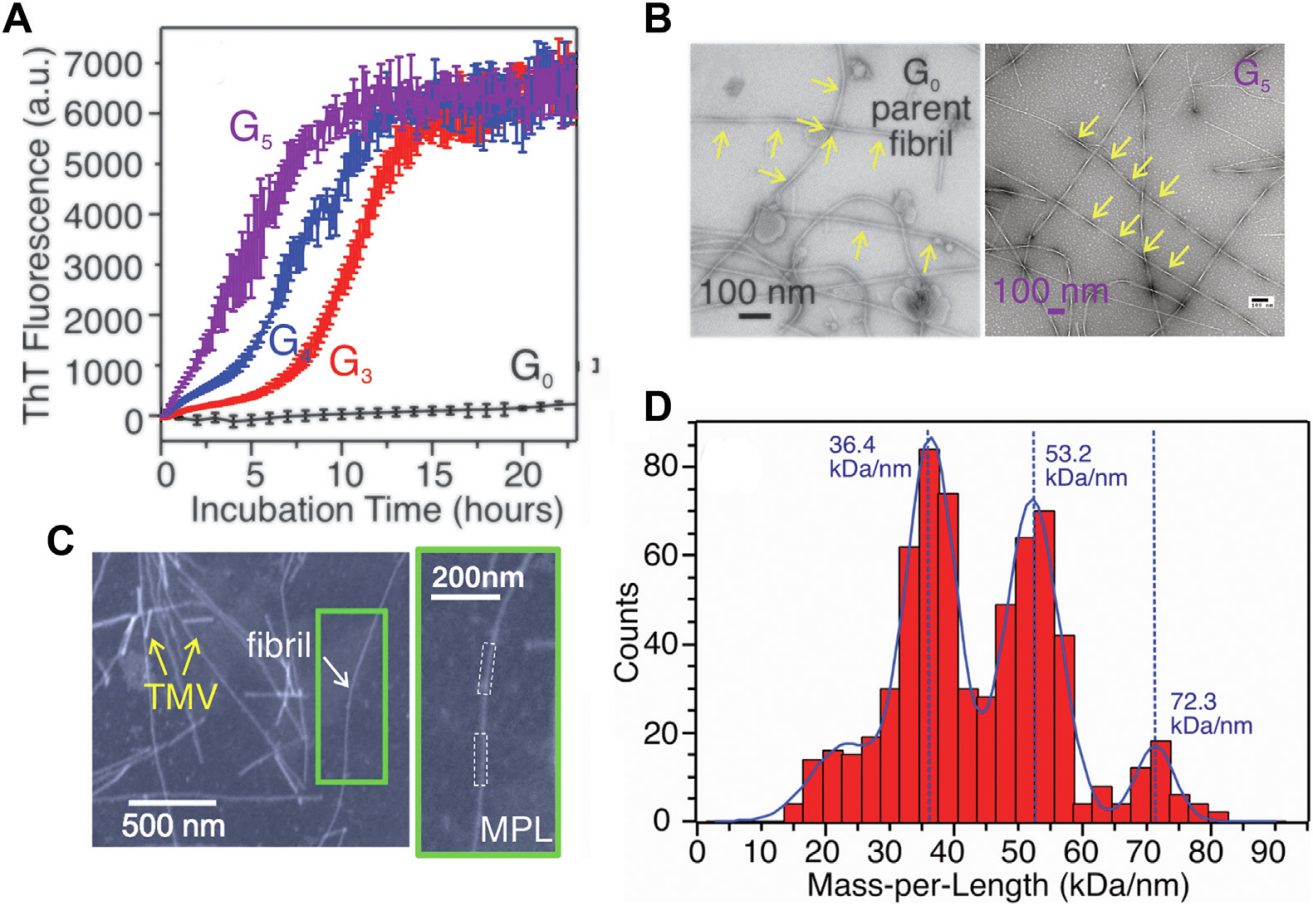
**Fluorescence and TEM characterization of rSPMs-Aβ**_**1–40**_**fibrils.***A*, ThT fluorescence kinetics for the parent G_0_ and seeded G_3_-G_5_ rSPMs-Aβ_1–40_ fibrils within the first 20 h incubation. Error bars were determined from 3 to 5 repetitions. *B*, negatively stained TEM images of G_0_ and G_5_ rSPMs-Aβ_1–40_ fibrils. *Yellow arrows* indicate the twisting morphological feature in both generations. *C*, dark-field TEM images for G_5_ rSPMs-Aβ_1–40_ fibril. Tobacco mosaic virus (TMV) rods (*yellow*) were utilized as internal standard. *D*, plot and fitting of the distribution of MPL obtained by analyzing ∼50 dark-field TEM images. Fitting indicates multiples of 2-fold symmetry. Aβ, β-amyloid; MPL, mass per length; ThT, thioflavin-T.

**Figure 5:**
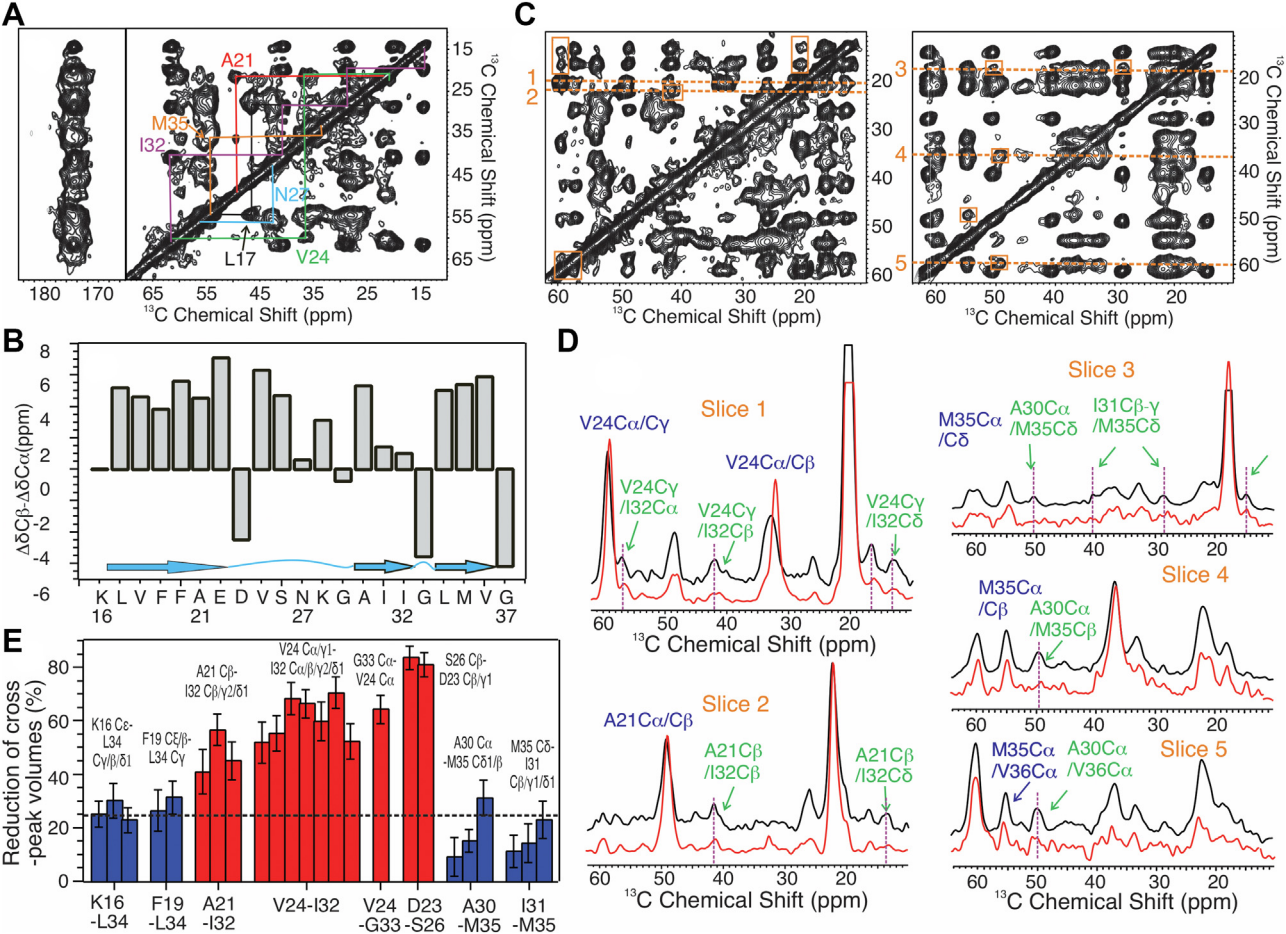
**Structural constraints from 2D**^**13**^**C-**^**13**^**C spin diffusion spectroscopy.***A*, a 2D spin diffusion spectrum with 20 ms mixing time (^13^C-uniform-labeling at G9, L17, A21, V24, N27, I32, and M35). Intraresidue crosspeaks are highlighted with *color lines*. *B*, plots of residue-specific ^13^C chemical shift deviations (ΔδC_β_ − ΔδC_α_). Positive deviations indicate β-strand secondary structures (*cyan arrows*). *C*, representative 2D spin diffusion spectra with 500 ms mixing time (*left*, sample with 100% isotope-labeled peptides; *right*, sample with 1:1 isotope dilution). Inter-residue crosspeaks are highlighted in *orange rectangles*. *D*, representative 1D slices along the chemical shifts shown by *dashed lines* in panel (*C*) (*black*, sample with 100% isotope-labeled peptides; *red*, sample with 1:1 isotope dilution). *E*, plot of isotope-dilution effects of inter-residue crosspeaks. Error bars were estimated from the signal-to-noise ratios from both nondiluted and diluted 2D spectra.

Using the parent Aβ_1–40_ fibril (G_0_) grown from 12 month rSPMs, we performed five-generation seeded fibrillation (G_1_-G_5_) to improve the homogeneity of final product, following previously developed protocols (53). Fibrillation time for generations G_3_-G_5_ was kept at 24 h to eliminate the self-nucleation from monomers (Fig. 4*A*). Mainly twisted fibrillar morphology was observed in both G_0_ and G_5_ fibrils with negatively stained transmission electron microscopy (TEM, *yellow arrows* in Fig. 4*B*). However, the G5 fibrils show higher tendency to form bundles (Fig. S6). This may be caused by repeated sonication during generation seeding, which tends to produce shorter and fragmentized filaments that are more likely to associate with each other. The mass per length (MPL) of G_5_ fibrils was determined using tilted-beam TEM (Fig. 4, *C* and *D*) (54), where distribution of MPL fit to multiples of ∼18.8 kDa/nm for typical Aβ_1–40_ fibrils with 2-fold rotational symmetry. Most individual measurements of MPL showed ∼36 and ∼54 kDa/nm, suggesting bundles of two and three filaments, respectively. 2D ^13^C/^13^C spin-diffusion spectroscopy (Figs. 5*A* and S7) led to ^13^C chemical shift assignments (Table S1). Residues A2 and G8-E11 do not show well-defined intraresidue crosspeaks, indicating disordered N terminus. ^13^C linewidths of K16-L17 and G37 are significantly broader compared with the segment V18-V36 (Fig. S8), suggesting that the latter segment forms the fibrillar core. Using the assigned residue-specific chemical shifts, TALOS+ predicts interruption of β-strand at the C-terminal residues G33-L34 (Fig. 5*B*).

Key inter-residue contacts were identified using long-range ^13^C/^13^C spin-diffusion spectroscopy. To characterize their intrastrand or interstrand nature, spectral were recorded with and without 1:1 isotope dilution. For the ones with dilution, 50% of Aβ_1–40_ peptides are unlabeled. Figure 5*C* (also see Fig. S9 and Table S3) show the 2D spectra with and without isotope dilution. Inter-residue contacts for pairs F19/L34, I32/A21, and D23/S26 are previously reported for Aβ_1–40_ fibrils (41, 42, 53, 54, 55, 56). However, contacts for pairs V24/I32, A30/V36, and I31/M35 are uncommon, indicating structural difference between the current rSPMs-Aβ_1–40_ fibril and reported Aβ_1–40_ fibrils grown in aqueous solutions. With 1:1 isotope dilution, the interstrand crosspeak intensities relative to their diagonal spin resources will reduce to ∼25% comparing to the nondiluted analogs, while the intrastrand crosspeak intensities are expected to be similar. Figure 5, *D* and *E* (also Table S3) shows that crosspeaks involving residues D23, V24, and S26 are intrastrand. Interactions involving the following residue pairs K16/L34, F19/L34, A21/I32, and A30-I31/M35-V36 are likely to be interstrand.

**Figure 6:**
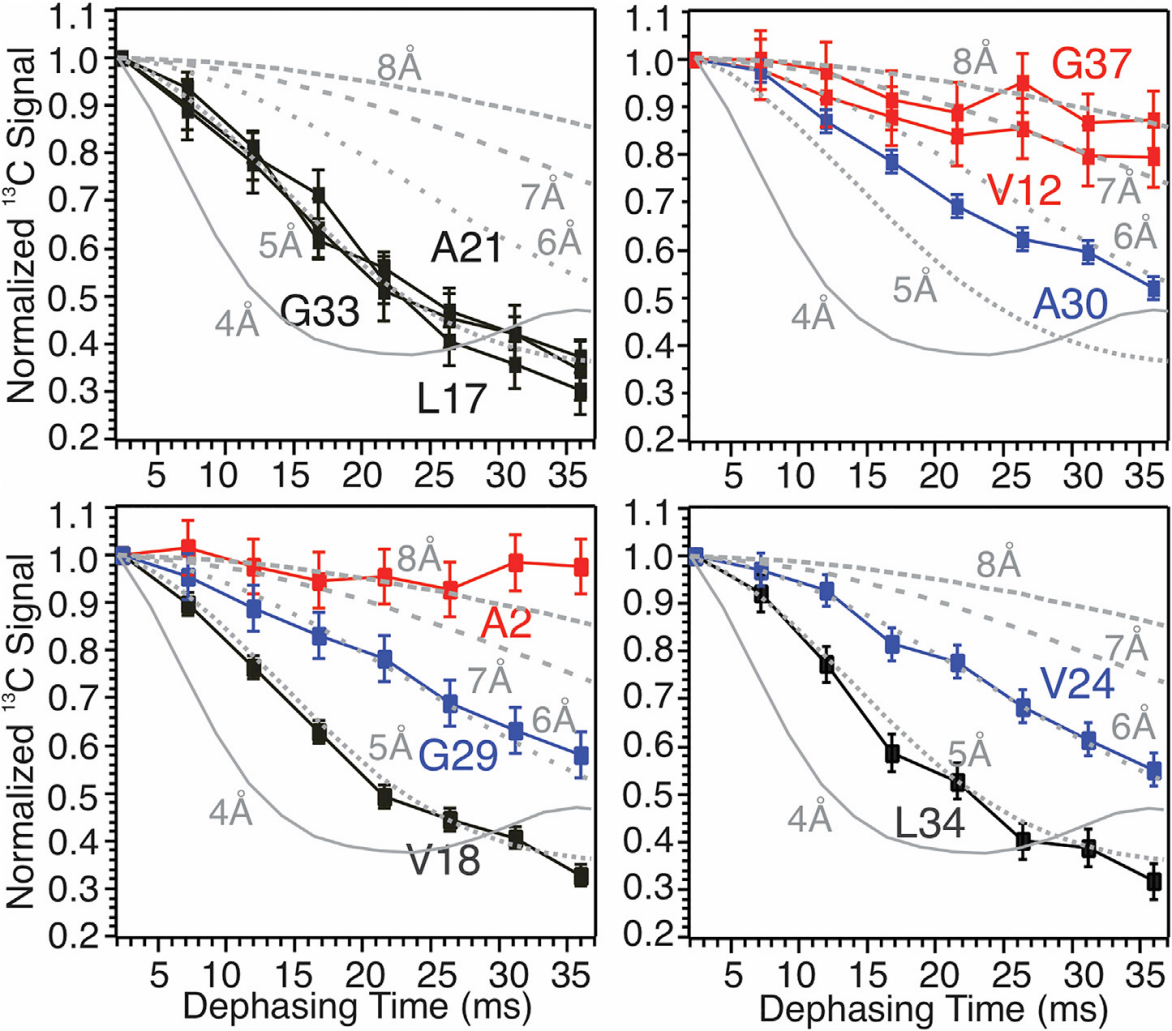
^**13**^**C-PITHIRDs-CT dephasing curves for the rSPMs-Aβ**_**1–40**_**fibril.** Individually isotope-labeled sites are given in panels. *Black*, *blue*, and *red colors* indicate residues with strong, medium, and little ^13^C dephasing, respectively. *Gray curves* represent simulated curves with 4 to 8 Å ^13^C-^13^C internuclear distances.

The rSPMs-Aβ_1–40_ fibrillar chain assembling was then probed using ^13^C-PITHIRDs-CT spectroscopy and singly isotope labeled sequences (Figs. 6 and S10; Table S2). Residues L17, V18, A21, G33, and L34 show typical rapid ^13^C signal decay for ∼5 Å interstrand distances. Considering the close side chain contacts observed in residue pairs A21/I32 and F19/L34, we excluded the possibility of antiparallel β-sheet backbone registry. Residues close to termini, such as A2, F4, and G37, show little ^13^C decay. This is consistent with the broadened ^13^C lines shown in 2D spectra and suggests disordered terminal structures. Interestingly, residues V24, G29, and A30 possess ^13^C signal decay curves that fit to ∼6 Å interstrand distance. Noting that residues within the same segment (*e.g.*, G25 and G29) reaches ∼6 Å interstrand distances at earlier nucleation stage (Fig. 2*C*), it is possible that these residues nucleate firstly through membrane-facilitated processes, and such initial aggregates contains interpeptide assemblies with parallel β-sheets that are not in registry.

### rSPMs-Aβ_1–40_ fibrils possess less compact hydrophobic core architecture, lower stability, and higher cytotoxicity

**Figure 7:**
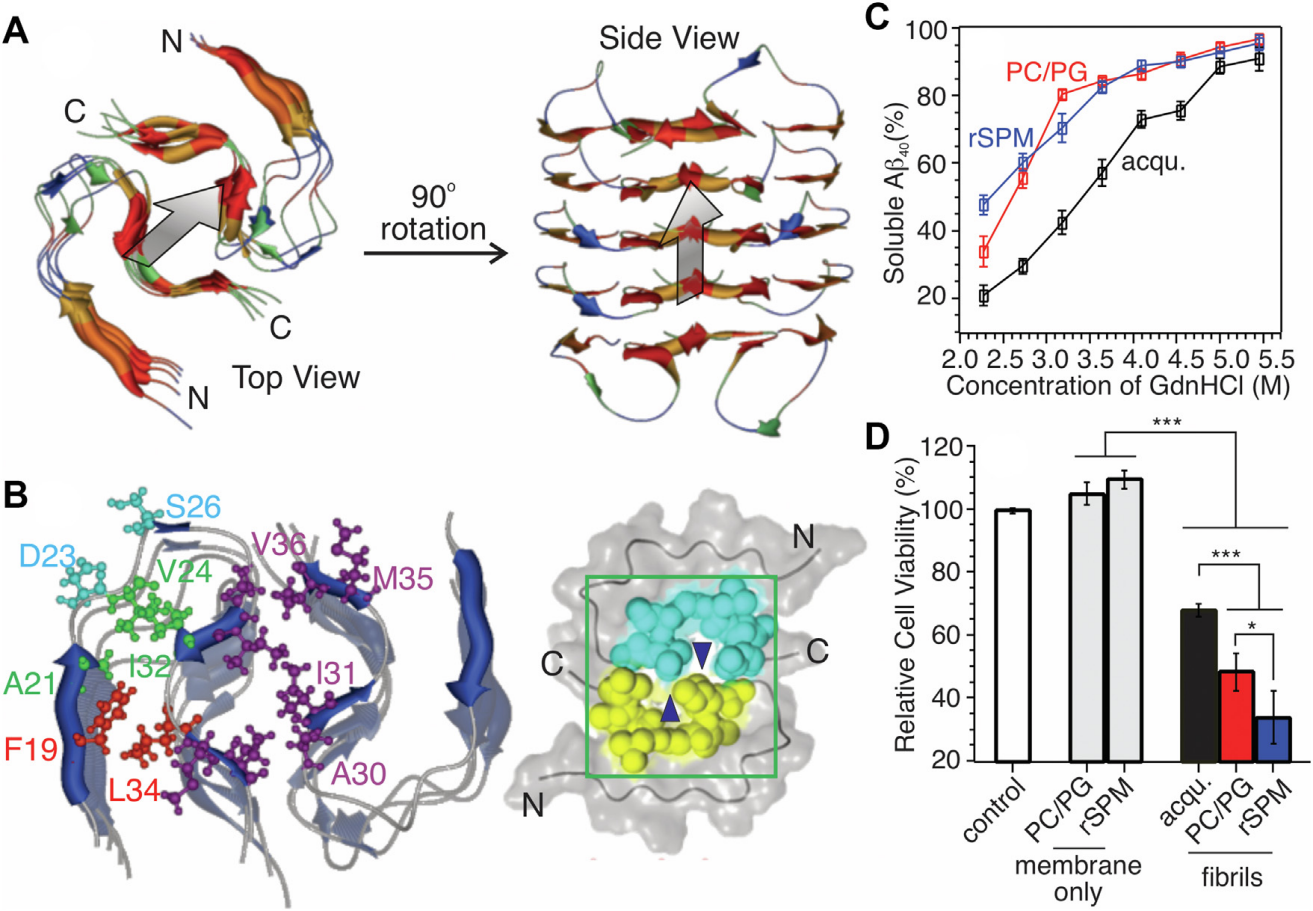
**Molecular structure, stability, and cytotoxicity of rSPMs-Aβ**_**1–40**_**fibril.***A*, the backbone architecture of rSPMs-Aβ_1–40_ fibrillar core segment K16-G37 (*left*, top view; *right*, side view). Residues are colored based on hydrophobicity (*red* for most nonpolar residues). *B*, sidechain contacts (*left*) and space-filling (right) models of rSPMs-Aβ_1–40_ fibrillar core. *Blue triangles* in *right panel* indicate hallows due to the C-terminal G33-L34 kink at quaternary interface. *C*, stability of various types of Aβ_1–40_ fibrils upon treating with GdnHCl. Error bars are derived from three repetitions. *D*, cell viability of N2a cells treated with various Aβ_1–40_ fibrils. Statistical significance is determined using standard *t* test based on 5 to 6 repetitions (∗*p* < 0.1; ∗∗∗*p* < 0.001). Color-coding in panels (*C*) and (*D*): *black*, aqueous Aβ_1–40_ fibril; *red*, Aβ_1–40_ fibrils grown with phosphatidylcholine/phosphatidylglycerol bilayer; *blue*, rSPMs-Aβ_1–40_. Aβ, β-amyloid.

A structural model (Fig. 7, *A* and *B*, using Xplor-NIH (57) package) for the core segment of rSPMs-Aβ_1–40_ fibril is established based on ssNMR constraints. Notably, the 2-fold quaternary interface is stabilized by interstrand contacts between A30/I31 and M35/V36 with a kink at G33-L34, supported by long-range 2D ^13^C/^13^C spin diffusion spectroscopy. This structural feature is distinct from most reported Aβ_1–40_ fibril structures formed in aqueous solutions, where uninterrupted C-terminal β sheets are commonly seen. As a result, the quaternary interfaces of aqueous Aβ_1–40_ fibrils are usually stabilized by stronger hydrophobic interactions involving a segment from I31 to V39. In the simulated lowest energy structures (Fig. S11), the backbone heavy atoms of segment D23-G29 possess larger RMSD value compared with the segments K16-E22 and A30-V36, in parallel with the PITHIRDs results where residues in this segment have longer interstrand distance and non parallel in-registry β-sheet structures.

The molecular structures of Aβ_1–40_ fibrils are known to be highly polymorphic and sensitive to the growth conditions. Notably, two Aβ_1–40_ fibrillar structures obtained under agitated growth conditions showed 2-fold symmetry in their quaternary interfaces (41, 58), while one structure with quiescent incubation showed 3-fold symmetry (40). Interestingly, the present rSPM-Aβ_1–40_ fibril forms under quiescent incubation yet shows 2-fold symmetry, indicating that the presence of membrane as an environmental factor modulate the molecular packing in the quaternary interface of Aβ_1–40_ fibrils. In addition, it is a common feature in all three aqueous Aβ_1–40_ fibrils that the β-sheets of C-terminal segment, that is I31-A40, are uninterrupted, which is different from the rSPM-Aβ_1–40_ fibrils. Recent ssNMR studies on Aβ_1–40_ fibrils seeded from Alzheimer’s brain tissue showed more wiggled C-terminal backbone structures (32, 42), suggesting that membrane-like environmental factors might influence Aβ fibrillation and fibrillar structural polymorphisms in Alzheimer’s disease patients.

Structural variations in membrane-associated Aβ_1–40_ fibrils may lead to distinct physicochemical and biological characteristics compared with aqueous Aβ_1–40_ fibrils. Stability of fibrils against guanidinium hydrochloride (GdnHCl) (59) is shown in Figure 7*C*. The results demonstrate that membrane-associated Aβ_1–40_ fibrils, grown from both rSPMs and a model phospholipid bilayer, are less stable compared with the aqueous Aβ_1–40_ fibril. The lower stabilities may be explained by their less compact quaternary interfaces, as it was previously shown that the hydrophobic fibrillar quaternary interface could be water accessible (60). Given that the G33-L34 kink at quaternary interface may result in “hallows” as shown in Figure 7*B*, the membrane-associated Aβ_1–40_ fibrillar assemblies would be more accessible to water-soluble GdnHCl. Figure 7*D* (and Fig. S12) shows the viabilities of neuroblastoma Neuro-2a (N2a) cells treated with Aβ_1–40_ fibrils grown in aqueous solution and various membrane models. The results indicate that membrane-associated fibrils possess higher cytotoxicity levels compared with the aqueous fibril, while the cytotoxicity difference between individual types of membrane associated fibrils is less significant. Previous studies have shown that the cytotoxicity levels of mature fibrils may correlate with their dynamic properties such as the elongation and shrinkage kinetics at fibril ends (61, 62, 63). Because these dynamic processes may cause more interruptive effects to cells comparing with inert mature fibrils. For instances, WT Aβ_1–40_ fibrils with larger shrinkage rates possess higher cytotoxicity levels (61). For the seeded fibrillation process, the posttranslationally modified Aβ fibrillar seeds with faster elongation rates led to higher levels of cytotoxicity (59, 64, 65). In the current work, the membrane-associated Aβ_1–40_ fibrils with less perfect quaternary interface hydrophobic interaction may possess more dynamic fibrillar ends.

## Discussion

**Figure 8:**
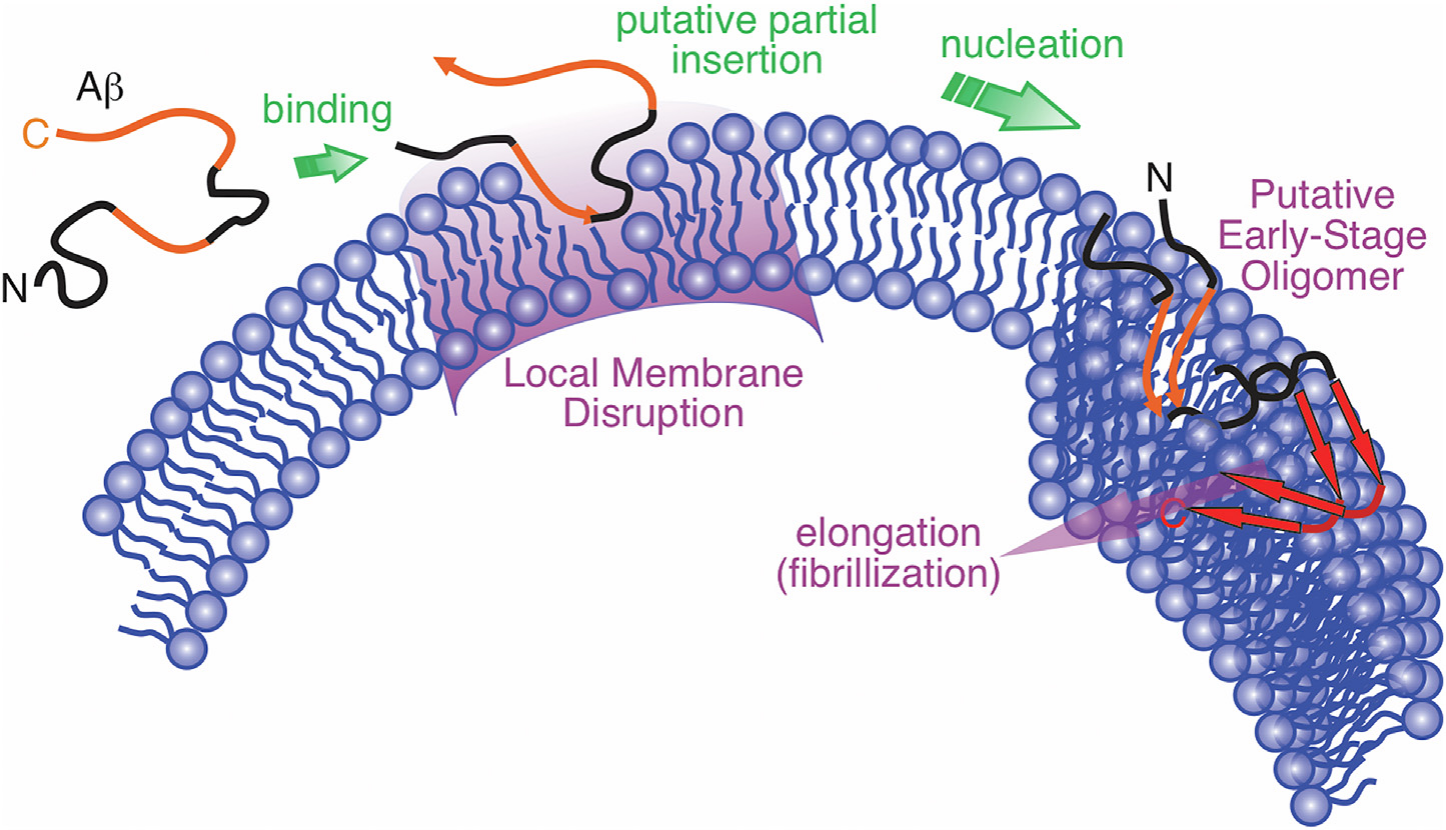
**Schematic illustration of membrane-associated Aβ**_**1–40**_**nucleation process from current experimental data.** Monomeric Aβ_1–40_ contains both polar (*black*) and nonpolar (*orange*) segments. Segment F19-G29 (*middle orange* and *black regions*) interact strongly with lipids at binding stage. Initial nucleation (β-sheet-like aggregation) occurs at the C-terminal G25-V36 segments of peptides. Aβ, β-amyloid.

We propose a schematic mechanism of Aβ_1–40_-lipid interactions that drive the membrane-associated nucleation process (Fig. 8). About 20% Aβ_1–40_ monomers, upon addition to rSPMs, are rapidly adsorbed to membrane surface and interact strongly with phospholipid headgroups, leading to restriction of lipid headgroup motions. The initial binding may be driven by electrostatic interactions involving polar N-terminal and F20-A30 segments of Aβ_1–40_, suggested by previous MD simulations (49, 66, 67). Membrane-bound Aβ peptides were shown to recruit new Aβ molecules from solution (43), supported by the increasing membrane-bound Aβ_1−40_ concentrations over the entire nucleation stage (*i.e.*, ∼20 h). Within the next 15 h, Aβ undergoes structural conversion to form nuclei for further fibrillation and the hydrophobic interactions are likely to serve as main driving force. Through the nucleation process, the C-terminal segment G25-V36 forms low order interstrand assemblies with β-strand backbone conformation but not necessarily the parallel β-sheet registry, supported by CD, FTIR, and PITHIRDs data. Segment F19-G25 shows close contact with phospholipids at the same time. We propose that hydrophobic interactions between the side chains within the segment (*e.g.*, F19, F20, A21, and/or V24) and the lipid alkyl chains strengthen the binding. Interactions with charged residues such as E22 and D23 may also be involved. Further dynamic nuclear polarization–-enhanced 2D ssNMR and heteronuclear dipolar coupling experiments are expected to address these questions. Consequently, lipid diffusive motion is restricted. The similar effect of lipid dynamics modulation was reported in a previous neutron scattering study using phosphocholine bilayer model (68).

Notably, the current rSPMs-Aβ_1–40_ fibril shares similar structural features with a previous model for Aβ_1–40_ fibrils grown in the presence of a model phosphatidylcholine/phosphatidylglycerol bilayer (28). Especially, the featured G33-L34 backbone kink is present in both models. Although the previous model does not include a quaternary interface, the side chains of I31 and V36 both orientate outward of intramolecular interface, which potential stabilize quaternary assemblies. These structural similarities suggest that a kinked and less compact C-terminal β-sheet segment may be a unique feature for membrane-associated Aβ_1–40_ fibrils, induced by common properties of membranes rather than specific components. Evidence from current PITHIRDs spectroscopy demonstrate that the C-terminal A30-V36 is the first segment to aggregate. There hence, its molecular structure is likely to be modulated by peptide-membrane interactions and propagate to mature fibrils. The time-dependent modulation of lipid diffusive motion (Fig. 3*C*) may provide insights on the structural transition. The rSPMs vesicles used in the current work are a few hundred nanometers in diameter. With this size, the value of τ_s_ is predominantly determined by the radius of lateral diffusive motion (44). Therefore, one possible explanation for the decrease of τ_s_ (*i.e.*, more rapid lateral diffusive motion) is the formation of highly curved edges within the bilayer through detergent-like effects (9, 22), which produce much smaller diffusion radius for a small population of lipids (as illustrated in Fig. 8). The aggregation of Aβ peptides may then occur at these edges where the curvature of membrane leads to the formation of G33-L34 kink and interrupted β-sheet in C-terminal segments.

One question to be answered is whether Aβ peptide molecules insert into and interrupt membrane bilayer at the nucleation stage. Our current and previous results lead to a hypothesis that Aβ forms low order β-sheet–enriched oligomers that insert and disrupt at least one leaflet of bilayer. In addition to the present CD, FTIR, and PITHIRDs results that support the predominant β-sheet structure, we previously showed using dynamic nuclear polarization–enhanced ssNMR that intrastrand interactions between F19 and L34 was present in Aβ_1–40_-membrane system within 8 h incubation (69). This means certain tertiary structures, such as β-hairpin, may also present in the early stage assemblies. In fact, Aβ_1–40_ protofilaments with intrastrand β-hairpin structures was recently identified in mature brain-seeded fibrils as well (32). Overall, with the satisfaction of backbone hydrogen bonding through intrastrand and/or interstrand interactions, low order Aβ oligomers with exposed nonpolar sidechains (*e.g.*, segment G25-V36) are more likely to intercalate into the bilayer interior rather than stay in polar phospholipid headgroup regions. Furthermore, instant membrane content leakage and lipid mixing have been observed when Aβ peptides were added externally to membrane bilayers (10, 13, 20), suggesting that at least local membrane interruption has been induced at the nucleation stage. However, we do not anticipate that Aβ forms transmembrane oligomers under the current condition, as those oligomers would eventually form membrane pore and/or induce membrane fragmentation and are considered off the pathway of fibrillation (5).

In summary, the present study reports early stage molecular interactions between Aβ_1–40_ peptides and lipids in biological rSPMs at the nucleation stage. ssNMR measurements demonstrate that the C-terminal segment G25-V36 initializes the interstrand assembling to form low order β-sheet structures and the segment F19-G25 associates closely with lipids. Hydrophobic interactions between Aβ_1–40_ and lipids modulate the phospholipid dynamics within the time frame of nucleation, leading to restriction of lateral diffusion and acceleration of lipid headgroup motions. The resultant rSPMs-Aβ_1–40_ fibrils show a unique molecular structure at the quaternary interface with interrupted C-terminal β sheets, which indicate a less compact core architecture compared with Aβ_1–40_ fibrils formed in aqueous solutions. The structural polymorphisms of rSPMs-Aβ_1–40_ fibril may influence its biologically relevant properties such as stability and cytotoxicity. The results provide molecular level insights on the early events of Aβ fibrillation in biological membrane environments.

## Experimental procedures

### Peptide synthesis

All Aβ_1−40_ peptides with isotope labeling at selected residues (listed in Table S2) were synthesized manually using routine solid-phase peptide synthesis protocols with 9-fluorenylmethyloxycarbonyl chemistry. The crude peptides were cleaved using a mixture of TFA/phenol/water/1,2-ethanedithiol/thioanisole with the volume ratio 90:5:10:5:2.5. All peptides were purified on a HPLC (HPLC 1260 Series, Agilent Inc) installed with C18 reversed-phase column, lyophilized, and stored at −20 °C freezers before usage. The purified peptides were verified with LC-MS/electrospray ionization (LCMS-2020, SHIMADZU Inc) to confirm >95% purity.

### Fibrillization of Aβ_40_ in the presence of rSPMs (parent G_0_ and seeded G_1_-G_5_)

The lyophilized A_1__4_ was dissolved in hexafluoro-isopropanol to a concentration of 2mg/ml and then bath-sonicated for 2 min to remove any preformed aggregates and then incubated in the hood overnight. The residual hexafluoro-isopropanol was removed by a gentle N_2_ gas flow followed by overnight treatment with high vacuum desiccator. The resulted peptide film was redissolved in dimethyl sulfoxide (DMSO) to 4 mM, the solution was centrifuged at 14,000 rpm for 10 min, and the supernatant was collected. The parent membrane associated Aβ_1−40_ fibril (G_0_) was generated by mixing the DMSO stock solution with the rSPMs stock solution. The molar ratio between Aβ_1−40_ peptides and the total lipids was kept at 1:30 and the Aβ_1−40_ concentration was 10 μM. The mixture was quiescently incubated at 37 °C for at least 1 week. The formation of G_0_ fibril was confirmed by negatively stained TEM and the increase of Th-T fluorescence intensity. For the generation seeding, 10% of G_0_ (relative to the total mass of monomeric Aβ_1−40_) was gently sonicated on ice-bath for 2 min using a probe sonicator (20% power output and 20% duty cycle) and then freshly pretreated Aβ_1−40_ in DMSO was added to 10 μM peptide concentration. The G_1_ fibrils were incubated quiescently for 48 h at 37 °C. Accordingly, the G_2_-G_5_ fibrils were produced using the same protocol as the G_1_ fibril except that the incubation time was kept at 24 h for each generation. About 4 mM Hepes buffer with 0.01% NaN_3_ (pH 7.4) was utilized for the fibrillation protocols.

### ThT fluorescence kinetics assay of Aβ_1−40_ fibrillization

ThT fluorescence assays were performed on Synergy HTX Multi-Mode Microplate Reader (BioTek Instruments, Inc) with excitation and emission wavelengths at 450 and 480 nm, respectively. Aliquots of 150 μl fibril solution were pipetted to the 96-well plate and the concentration of the ThT solution (dissolved in 4 mM Hepes buffer pH 7.4, 0.01% NaN_3_) was kept at 50 μM. The kinetic measurements were performed at 37 °C and samples were mixed for 10 s before the data points were collected. Three repetitions were performed for fibrillation with each membrane compositions and in the aqueous solutions.

### BCA assay

The BCA assays (standard kits, Sigma–Aldrich Inc) were used to quantify the binding percentage of Aβ_1–40_ to rSPMs, following the published protocols by McArdle *et al.* (70). The samples and their corresponding controls containing the same concentrations of rSPMs without the addition of Aβ_1–40_ were incubated for different designed time periods and ultracentrifuged at 50,000 rpm (on a Beckman Coulter Ultracentrifuge with a TLA-110 rotor, 4 °C). The supernatants were collected and mixed with 0.2 M NaOH containing 2% (w/v) SDS. The mixtures were heated to 95 °C for 5 min. After cooling to ambient temperature, 25 μl of the supernatant was added to the microplate wells followed by adding 200 μl of BCA working reagents (Reagent A:B = 50:1 (*v/v*)). The plates were sealed and incubated at 37 °C for 30 min on a well-plate reader (BioTek Synergy HTX, BioTek Inc). The plate was cooled down to room temperature (RT) for 10 min and the absorbance at 560 nm was measured. Three repetitions were performed on each membrane conditions and incubation times. The concentrations of Aβ_1–40_ peptides were determined based on the standard curve obtained on bovine serum albumin, and the binding percentages of Aβ_1–40_ peptide to rSPMs were determined as follows:

$$\text{A}{\text{β}}_{1-40}^{Membrane-bound\;}\text{\%}=\frac{C_{supernatant\;with\;A\beta_{40}\left(sample\right)}-C_{supernatant\;without\;A\beta_{40}\left(control\right)}}{C_{initial\;A\beta_{40}}}*100\%$$

### CD spectroscopy

CD spectra were collected on a JASCO-J-1100 spectrophotometer from 190 nm to 260 nm with 20 scans of signal averaging. Aβ_1–40_ peptides were added to rSPMs and incubated for different time periods and centrifuged (Beckmann Coulter Benchtop ultracentrifuge) for 30 min at 50,000 rpm. Supernatant and liposome pellet were separated, and the pellet was resuspended in the same volume of buffer. Both supernatant and resuspended pellet were analyzed by CD. All spectra were analyzed using CDPro software (JASCO Inc) to obtain the distribution of secondary structures.

### FTIR spectroscopy

Attenuated total reflectance-FTIR) spectra were recorded on a Spectrum Two FTIR spectrometer from 4000 cm^−1^ to 400 cm^−1^. The out-of-compartment, horizontal attenuated total reflectance accessory was incorporated to permit analysis of large samples of low peptide concentration of 10 μM. Sixty-four accumulations were performed to improve the signal/noise ratio. Spectra were recorded at a resolution of 4 cm^−1^. All measurements were made at RT 24 °C. The liposome was prepared by dissolving the total lipid brain extract (purchased from Avanti Polar Lipids) in chloroform and obtaining the dried lipid film under N_2_ flow and high -vacuum desiccator. The lipid was resuspended in 20 mM phosphate buffer (pH 7.4, 150 mM NaCl, 0.01% NaN_3_). Ten freeze and thaw cycles using liquid N_2_ and water-bath sonicator followed by 30 cycles of extrusion with 0.4 μM pore size membranes were used to obtain the large unilamellar vesicles. The peptide was added with concentration of 10 μM, and the sample incubated for different time points then centrifuged. Both supernatant and resuspended pellet were analyzed by spreading 300 μl of the solution on the trough plate and absorbance data recorded for further data analysis.

### Negatively stained TEM and MPL measurements

Negatively stained TEM was utilized to observe the morphology of fibrils. Briefly, a 10 μl aliquot of fibril solution was placed on a glow-discharged carbon film, supported by lacey carbon on a copper TEM grid (300 mesh; Ted Pella Inc) and adsorbed for 2 min. The grid was blotted, rinsed with 10 ml of deionized water, blotted, rinsed again, blotted, then stained with 2% uranyl acetate for 30 s, blotted, and dried in air. TEM images were recorded on a FEI Morgagni microscope, operating at 80 kV, equipped with a side-mounted Advantage HR camera (Advanced Microscopy Techniques). Images were recorded with 44,000× to 89,000× magnifications. Dark-field TEM images of an unstained grid were recorded for MPL measurements, as previously described. To analyze the MPL, integrations of intensities were measured for selected regions in fibrils, the internal standard tobacco mosaic virus (TMV), and the background of TEM grid. Over 500 segments on fibrils were analyzed from ∼60 dark-field TEM images to generate the histograms shown in Figure 1, where individual MPL value was calculated using the following equation:

$$MPL=\frac{I_{TMV}-I_{B,\;TMV}}{I_{Fibril}-I_{B,Fibril}}\times 131kDa/nm,$$

where *I*_*TMV*_ and *I*_*Fibril*_ were integrated intensities in defined areas on TMV and fibrils, and *I*_*B,*__*TMV*_ and *I*_*B,Fibril*_ were integrated intensities for the size of areas of background adjacent to the selected TMV and fibrils respectively.

### Cytotoxicity of Aβ_1−40_ fibrils in neuroblastoma N2a cells

The N2a cells were thawed in 37 °C water bath and centrifuged to remove the supernatant at 2000 rpm for 2 min. The pellets were resuspended in fresh growth medium and cultured under standard conditions at 37 °C in humidified atmosphere (95% humidity, 5% CO_2_). After reaching 90% confluency, the cells were trypsinized and passaged for the growth of new generation with a ratio of 1:10 (*v/v*). The cells were harvested after five generations of passaging and around 7500 cells were loaded to each one of wells on a 96-well plate (Corning Inc) with corresponding controls (membranes only) and samples (membranes with Aβ_40_ fibrils). Fibrils utilized in the N2a cell viability studies were preformed in the presence of various model membranes and incubated for at least 14 days. Formation of mature fibrils was confirmed by ThT fluorescence assay and CD spectroscopy as described in previous sections. Both controls and samples were sonicated on ice for 150 s before the addition to cells. After 24 h incubation, the original growth medium was carefully removed, and then 100 μl of fresh growth medium and 10 μl of 12 mM filtered 3-(4,5-dimethylthiazol-2-yl)-2,5-diphenyltetrazolium bromide solution were added to each well, which were further incubated for 4 h. For detection, 85 μl of the cell culture were gently taken, followed by the addition of 100 μl DMSO. The mixture was then incubated again for 10 min at 37 °C and the absorbances were measured on a Synergy |HTX Multi-Mode Microplate Reader (BioTek Instruments, Inc) at 570 nm. The percentages of cell viability were calculated by the following equation:

$$Cell\;viability\;\%=\frac{A_{sample}-A_{background}}{A_{control}-A_{background}}\times 100\%$$

where $A_{sample}$ is the absorbance of the sample after treated with Aβ_1−40_ fibril, $A_{background}$ is the absorbance of DMSO, and $A_{control}$ is the absorbance of the cell with the addition of membranes in the absence of Aβ_1−40_ fibrils. Five to six repetitions were performed on each sample condition.

### Stability of Aβ_1−40_ fibrils measured by GdnHCl denaturing assays

In the present study, GdnHCl was applied to assess the stabilities of fibrils grown from the isolated synaptic plasma membranes, the aqueous buffer, and the model phospholipid bilayer. The lyophilized fibrils were resuspended in 4 mM Hepes buffer (0.01% NaN_3_, pH 7.4) to a concentration of 46.2 μM (estimated based on the initial concentration of Aβ_40_ peptides) and then ice-bath sonicated for 30 min to obtain a homogeneous solution. Two hundred microliters aliquots of the aforementioned fibril solution were mixed with 2 ml GdnHCl solutions with various concentrations ranging from 2.5 M to 6 M. The final concentrations of GdnHCl were from 2.27 M to 5.46 M. The mixtures were incubated at 37 °C for 36 h with gentle shaking at 120 rpm. After incubation, the mixtures were centrifuged at 95,000 rpm (Beckman ultracentrifuge, TLA 100.4) at 4 °C for 1 h. The pellets were collected, washed with deionized water, and recentrifuged. The final pellets were resuspended in 200 μl deionized water and sonicated for 2 min on ice by a 450 W ultrasonic homogenizer sonicator (Vevor, 20% power level). Twenty-five microliters of the aforementioned solutions was added to the microplate wells with 200 μl of BCA working reagents (Thermo Fisher Inc). The plate was covered, incubated at 37 °C for 1 h, cooled to RT for 10 min, and then the absorbance was measured at 560 nm. Three repetitions were performed on each concentration of GdnHCl and fibrillation conditions.

### The ssNMR spectroscopy

All ssNMR measurements described in the current work were done on a Bruker 14.1 T spectrometer equipped with a Tri-Gamma magic angle spinning (MAS) probe tuned to ^1^H, ^31^P, and ^13^C resonance frequencies. For 2D ^13^C-^13^C spin diffusion, ^13^C-PITHIRDs-CT and ^13^C-^31^P REDOR measurements, sample temperature was kept at ∼280 K by monitoring the ^1^H frequencies in H_2_O. For ^31^P relaxation spectroscopy, several temperatures between 278 K and 296 K were utilized. The 2D ^13^C-^13^C spin diffusion spectroscopy was performed with the following parameters: a 70 kHz ^1^H π/2 pulse, a 50 kHz ^13^C crosspolarization (CP) field with linear ramp, and 50 kHz ^13^C pulses and 10 kHz radiofrequency-assisted diffusion for 20 ms and 500 ms for detecting the intraresidue and inter-residue interactions, respectively. The MAS frequency was kept at 10,000 ± 2 Hz. Typical signal-averaging time was 12 to 24 h for short-mixing experiments and 24 to 48 h for long-mixing experiments. The ^13^C-PITHIRDs-CT spectroscopy was performed at 20,000 ± 2 Hz MAS frequency, the same CP condition as the spin-diffusion spectroscopy and 30 kHz rotor-synchronized ^13^C π pulses. The spectrometer transmitter was adjusted to be on the resonance of carbonyl, alpha, and methyl carbons for individual labeling sites. The ^13^C-^31^P REDOR spectroscopy utilized the same CP condition, 45 kHz rotor-synchronized ^31^P p pulses, and 8 kHz MAS frequency. Pulsed-spin locking acquisition algorithm was applied for both PITHIRDs-CT and REDOR spectroscopy to enhance the signal-to-noise ratio. For ^31^P relaxation spectroscopy, the MAS frequency was kept at 10 kHz with 56 kHz ^31^P π/2 and π pulses.

### Structural modeling

Atomic coordinates for a 10-molecule segment of the membrane associated Aβ_40_ fibril was generated by restraint MD and simulated annealing using Xplor-NIH package. Potential energy functions representing distance restraints from 2D spin-diffusion and ^13^C-PITHIRDs-CT spectroscopy, as well as the backbone dihedral angle restraints derived from ^13^C chemical shifts were applied. Standard bond length, bond angle, improper dihedral, and nonbonded repulsive potentials were utilized. In addition, “residual dipolar coupling (RDC)” and interstrand O-H_N_ and O-C distance restraints were added for residues L17-A21, which were determined to be in parallel β-sheets based on chemical shifts and ^13^C-PITHIRDs data.

Structural modeling runs began with ten copies of Aβ_40_ sequence in an extended conformation in two layers (five copies per layer), placed with 40 Å center-of-mass spacings between peptides within one layer and 50 Å center-of-mass spacing between layers. A brief period of high temperature dynamics (2500 K, 10,000 simulation steps) was performed with only dihedral angle potentials (scaling factor 5). Annealing was then carried out in two stages. The first stage simulation annealing was from 2500 K to 1000 K with 5 × 10^6^ total steps, with distances (scaling factors ramped from 1 to 10) and dihedral angle potentials (constant scaling factor at 5), but without RDC potentials. The second stage simulation annealing was from 1000 K to 10 K with 6.5 × 10^6^ total steps with the application of dihedral angle potentials (constant scaling factor at 5), distance (ramped from 10 to 20) and RDC potentials (ramped from 1 to 5). Energy minimization was performed after second stage annealing. Twenty Xplor-NIH runs were performed, obtaining seven structures without violations in distances (within 0.5 Å threshold). The lowest energy structure was selected as the initial coordinate for a second iteration of the two-step annealing process, where all parameters were kept the same as except for the temperature gradient. The first stage annealing took temperature ramp from 1500 to 500 K and the second stage was from 500 to 20 K. Another 20 runs were performed and eight structures with the lowest restraint energies were reported. Total target energies range from 112.41 to 119.92 in Xplor-NIH units. No violations of bond angles, bond lengths, improper angles, or RDC restraints were reported for the final structure. No violation in distance restraints was reported for the eight lowest energy structures. An average 42.0 ± 3.8 violations for the dihedral angle restraints were reported out of a total 440 restraints with the maximum violation ∼32°.

### Quantitative analysis of lipid dynamics and 2D spin diffusion with isotope dilution

Quantitative analysis was done to the ^31^P relaxation spectroscopy to derive the values of τ_f_ and τ_s_. The ^31^P resonance peaks in T_1_ or T_2_ spectra were integrated over a 1.0 ppm range and the normalized peak volumes were plotted as a function of delay times and fit to exponential functions for T_1_ or T_2_ relaxation to obtain the decay time constants τ_1_ or τ_2_, respectively:

$$I\left(t\right)=I_{0}\;\exp\left(-t/\tau_{2}\right)\text{for T}2$$

$$I\left(t\right)=I_{0}-2I_{0}\;\exp\left(-t/\tau_{1}\right)\text{for T}1$$

The rate constant for the T_1_ and T_2_ decay, denoted as R_1_ and R_2_ respectively:

$$R_{1}=1/\tau_{1}$$

$$R_{2}=1/\tau_{2}$$

The correlation times are related to the relaxation decay rate constants according to the following quadratic equations:

$$R_{1}=\frac{2}{15}\omega_{31P}^{2}\sigma^{2}\left(1+\frac{\eta^{2}}{3}\right)\left[\frac{S^{2}\tau_{s}}{1+{\left(\omega_{31P}\tau_{s}\right)}^{2}}+\frac{\left(1-S^{2}\right)\tau_{f}}{1+{\left(\omega_{31P}\tau_{f}\right)}^{2}}\right]$$

$$R_{2}=\frac{1}{15}\omega_{31P}^{2}\sigma^{2}\left(1+\frac{\eta^{2}}{3}\right)\left\{\left[\frac{S^{2}\tau_{s}}{1+{\left(\omega_{31P}\tau_{s}\right)}^{2}}+\frac{\left(1-S^{2}\right)\tau_{f}}{1+{\left(\omega_{31P}\tau_{f}\right)}^{2}}\right]+\frac{4}{3}\left[s^{2}\tau_{s}+\left(1-S^{2}\right)\tau_{f}\right]\right\}$$

Approximation was made based on the magnitude of the terms in these equations to simplify the calculation. We consider the orders of magnitude of τ_s_ (∼10^−9^), τ_f_ (∼10^−6^), and the values of constants in the quadratic equations, where $\omega_{31P}$, σ, and η were the spectrometer frequency, the chemical shift anisotropy, and the asymmetric parameter of ^31^P, respectively, and had the values 2π×242MHz, 160 ppm, and 0.57. The order parameters *S* was estimated as 0.2 based on previous studies using lipid bilayer models with biologically relevant compositions. The first terms in both R_1_ and R_2_ are neglectable because they are ∼10^−15^ while all other terms are between 10^−8^ and 10^−10^. Furthermore, ${\left(\omega_{31P}\tau_{f}\right)}^{2}$ is ∼4 to 10 considering the approximate range of $\tau_{f}$ from all samples and constant $\omega_{31P}$ and is considerably larger than 1. Therefore, the second terms in both R_1_ and R_2_ are estimated as $\frac{2}{15}\sigma^{2}\left(1+\frac{\eta^{2}}{3}\right)\left(1-S^{2}\right)\tau_{f}^{-1}$ and $\frac{1}{15}\sigma^{2}\left(1+\frac{\eta^{2}}{3}\right)\left(1-S^{2}\right)\tau_{f}^{-1}$, respectively. The quadratic equations are therefore estimated as follows:

$$R_{1}=\frac{2}{15}\sigma^{2}\left(1+\frac{\eta^{2}}{3}\right)\left(1-S^{2}\right)\tau_{f}^{-1}$$

$$R_{2}=\frac{1}{15}\sigma^{2}\left(1+\frac{\eta^{2}}{3}\right)\left(1-S^{2}\right)\tau_{f}^{-1}+\frac{4}{45}\omega_{31P}^{2}\sigma^{2}\left(1+\frac{\eta^{2}}{3}\right)\left[s^{2}\tau_{s}+\left(1-S^{2}\right)\tau_{f}\right]$$

For samples containing Aβ_1–40_ and controls without the presence of peptides and the same incubation time, the values of $\tau_{f}$ and $\tau_{s}$ were calculated using these simplified equations. The difference between samples and their corresponding controls were plotted in Figure 4, *B* and *C*.

The propagated uncertainties of $\tau_{s}$ and $\tau_{f}$ were calculated as follows:

$$\sigma_{\tau_{f}}=\frac{C1}{\omega_{31P}^{2}}\sigma_{T_{1}}$$

$$\sigma_{\tau_{s}}=\sqrt{{\left(\frac{-C2}{T_{2}^{2}}\right)}^{2}\sigma_{T_{2}}^{2}+{\left(\frac{C3}{T_{1}^{2}}-C4\right)}^{2}\sigma_{T_{1}}^{2}},$$

where C1–C4 are constants derived from the ^31^P Larmor frequency, the CSA, the asymmetry parameter, and the order parameters, and their values are $8.74\times 10^{9}$, $4.29\times 10^{-9}$, $2.20\times 10^{-9}$, and $9.58\times 10^{-8}$, respectively.

To quantify the isotope-dilution effect, the peak volumes for a specific inter-residue crosspeak in the nondiluted and diluted spectra were obtained by integrating over a 1.5 × 1.5 ppm spectral region around the peak center and denoted $V_{1,n}$ and $V_{1,d}$, respectively. Similarly, their corresponding diagonal peaks (serves as references) were integrated and denoted as $V_{0,n}$ and $V_{0,d}$, respectively. The spectral noises were determined as the SDs of the volumes of 10 different 1.5 × 1.5 ppm spectral regions without peaks and denoted as $\delta_{n}$ and $\delta_{d}$, respectively. With these denotations, the reduction of crosspeak volumes (the *y*-axis in Fig. 6*E*) was calculated as follows:

$$Reduction=\left(\frac{V_{1,d}}{V_{0,d}}\right)/\left(\frac{V_{1,n}}{V_{0,n}}\right)\times 100\%$$

The uncertainty of the reduction of cross peak volumes (the error bars in Fig. 6*E*) was propagated as follows:

$$Uncertainty\;of\;Reduction=\sqrt{{\left(\frac{V_{1,d}}{V_{0,d}V_{1,n}}\right)}^{2}\cdot \delta_{n}^{2}+{\left(\frac{V_{0,n}}{V_{0,d}V_{1,n}}\right)}^{2}\cdot \delta_{d}^{2}+{\left(\frac{-V_{1,d}V_{0,n}}{V_{0,d}{V_{1,n}}^{2}}\right)}^{2}\cdot \delta_{n}^{2}+{\left(\frac{-V_{1,d}V_{0,n}}{V_{1,n}{V_{0,d}}^{2}}\right)}^{2}\cdot \delta_{d}^{2}}$$

### Statistical analysis

Uncertainties of measured parameters, including the lag periods of ThT fluorescence kinetics traces, the membrane-bound Aβ_1–40_ concentrations determined by BCA assay, the secondary population distributions derived from CD spectra, the correlation times derived from the ^31^P relaxation spectroscopy, and the concentration of denatured Aβ_1–40_ in GdnHCl assay, were determined by calculating the SDs of corresponding measurements in 3 to 5 independent repetitions. The statistical significance values between individual sample groups in the 3-(4,5-dimethylthiazol-2-yl)-2,5-diphenyltetrazolium bromide cell viability assay were determined by standard *t* test.

## Data availability

All data are contained within the article.

## Supporting information

This article contains supporting information.

## Conflicts of interest

The authors declare that they have no conflicts of interest with the contents of this article.
