# Supplementary material for: Early stage β-amyloid-membrane interactions modulate lipid dynamics and influence structural interfaces and fibrillation
Source: J Biol Chem. 2022 Sep 14;298(10):102491. doi: 10.1016/j.jbc.2022.102491 (PMC9556791; doi:10.1016/j.jbc.2022.102491)
Supplement: Supplemental Figures S1–S12 and Tables S1–S3 [file mmc1.docx]

**Supporting Information**

**Early-Stage β-Amyloid-Membrane Interactions Modulate Lipid Dynamics and Influence Structural Interfaces and Fibrillation**

June M. Kenyaga, Qinghui Cheng and Wei Qiang

Department of Chemistry, Binghamton University, the State University of New York

***S.I. Figures***

**
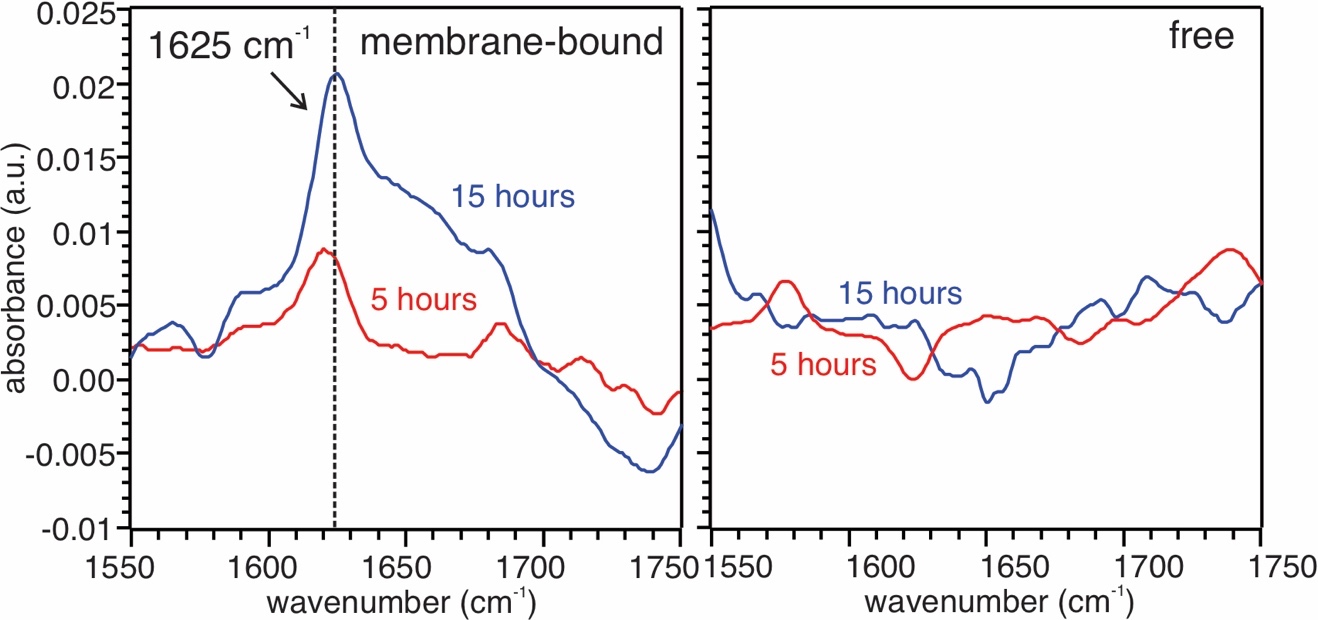
**

**Figure S1** ATR-FTIR analysis of the time evolution of membrane-bound (left panel) and free (right panel) Aβ_1-40_ secondary structures. Spectra with short incubation time were subtracted from the spectra with 5 and 15-hour incubation to obtain the above difference spectra. Peaks at 1625 cm^-1^ indicate typical parallel β-sheet in amide-I band for C=O stretching.


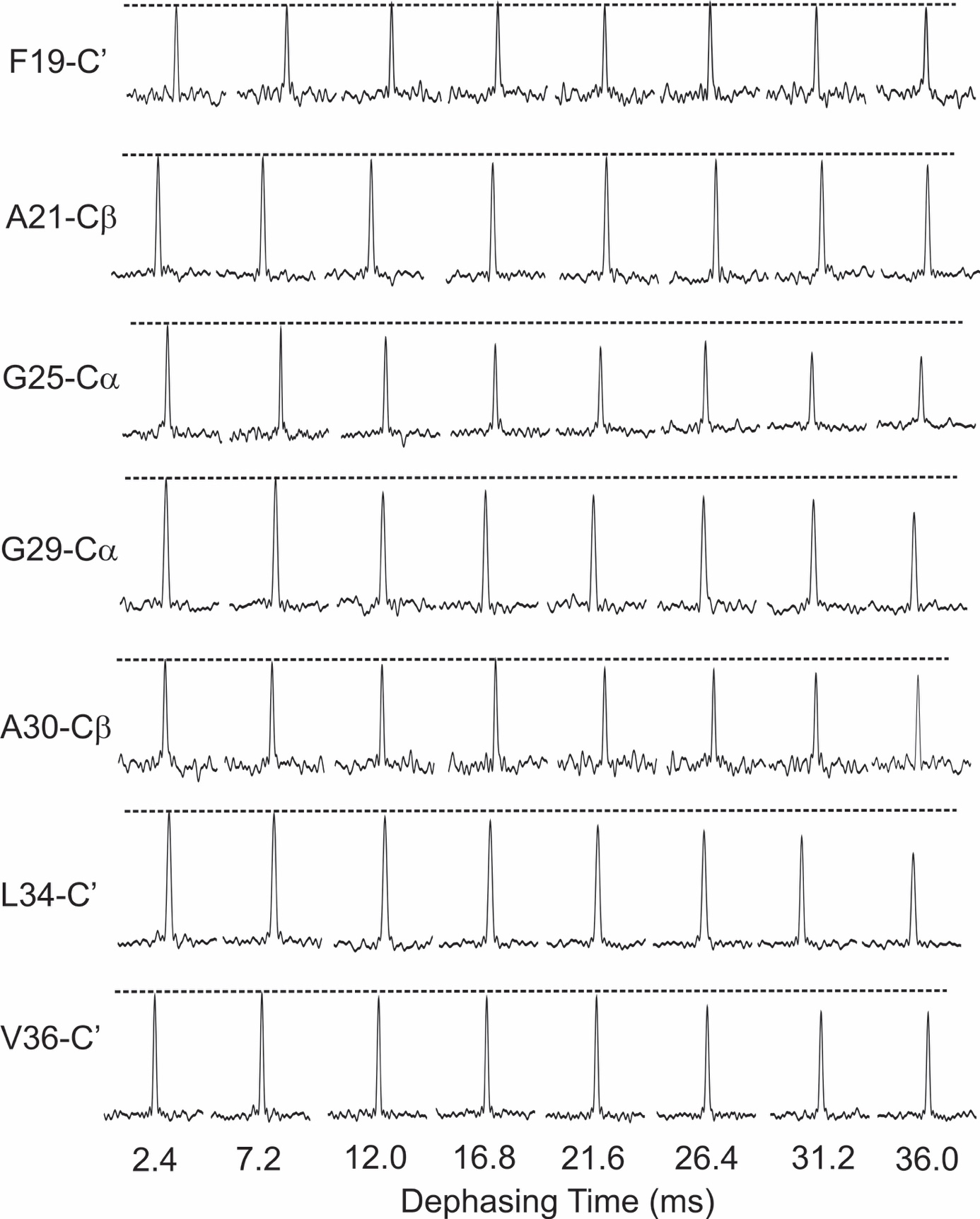


**Figure S2** All ^13^C-PITHIRDs-CT spectra for individual isotope-labeled sites with 5-hour incubation time periods. All spectra were collected with pulsed spin locking acquisition algorithm so that the transmitter was moved to the targeting resonance peak. Therefore, no chemical shift information was obtained.


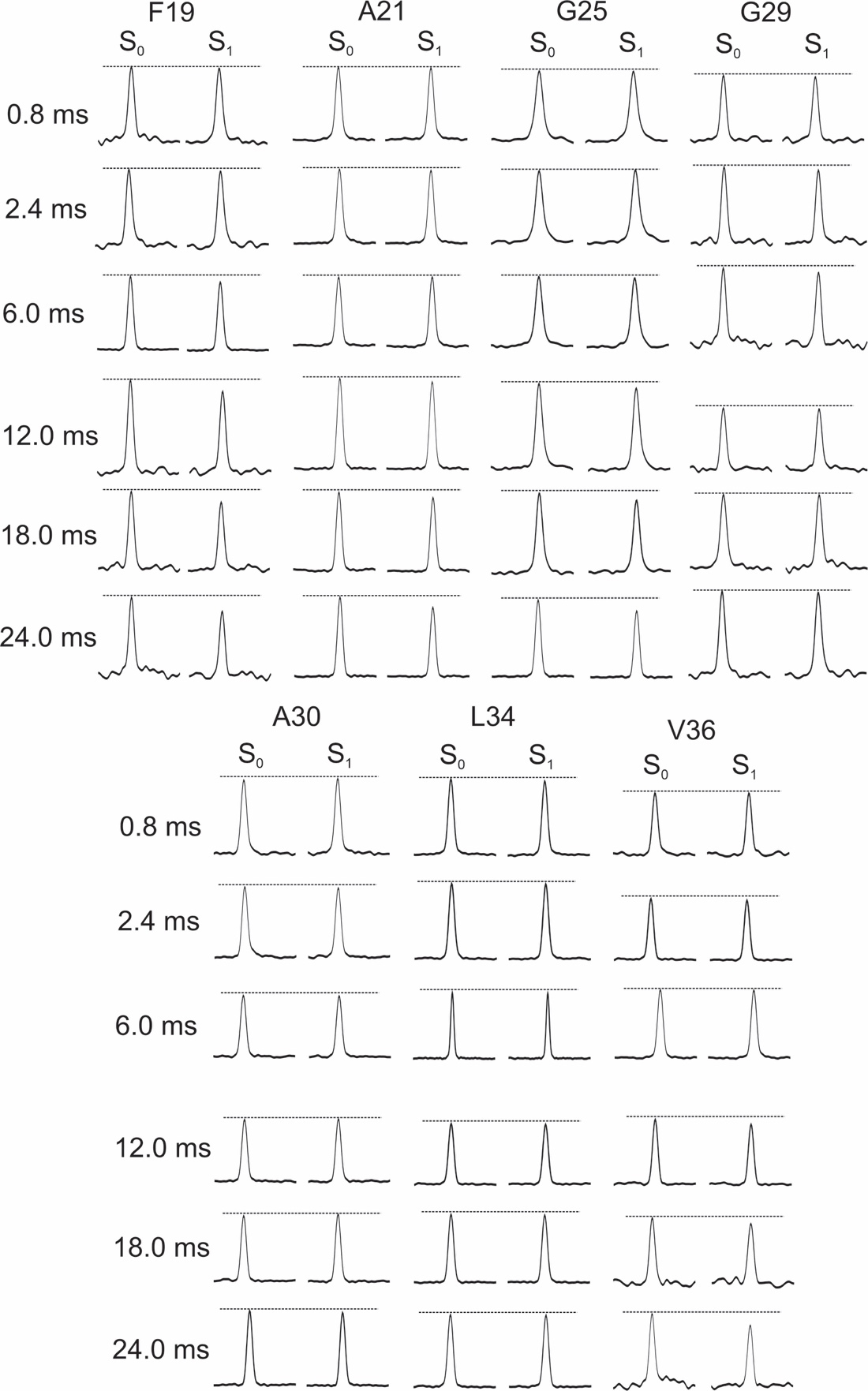


**Figure S3** All ^13^C-^31^P REDOR spectra for individual isotope-labeled sites with 5-hour incubation time periods. All spectra were collected with pulsed spin locking acquisition algorithm so that the transmitter was moved to the targeting resonance peak. Therefore, no chemical shift information was obtained.


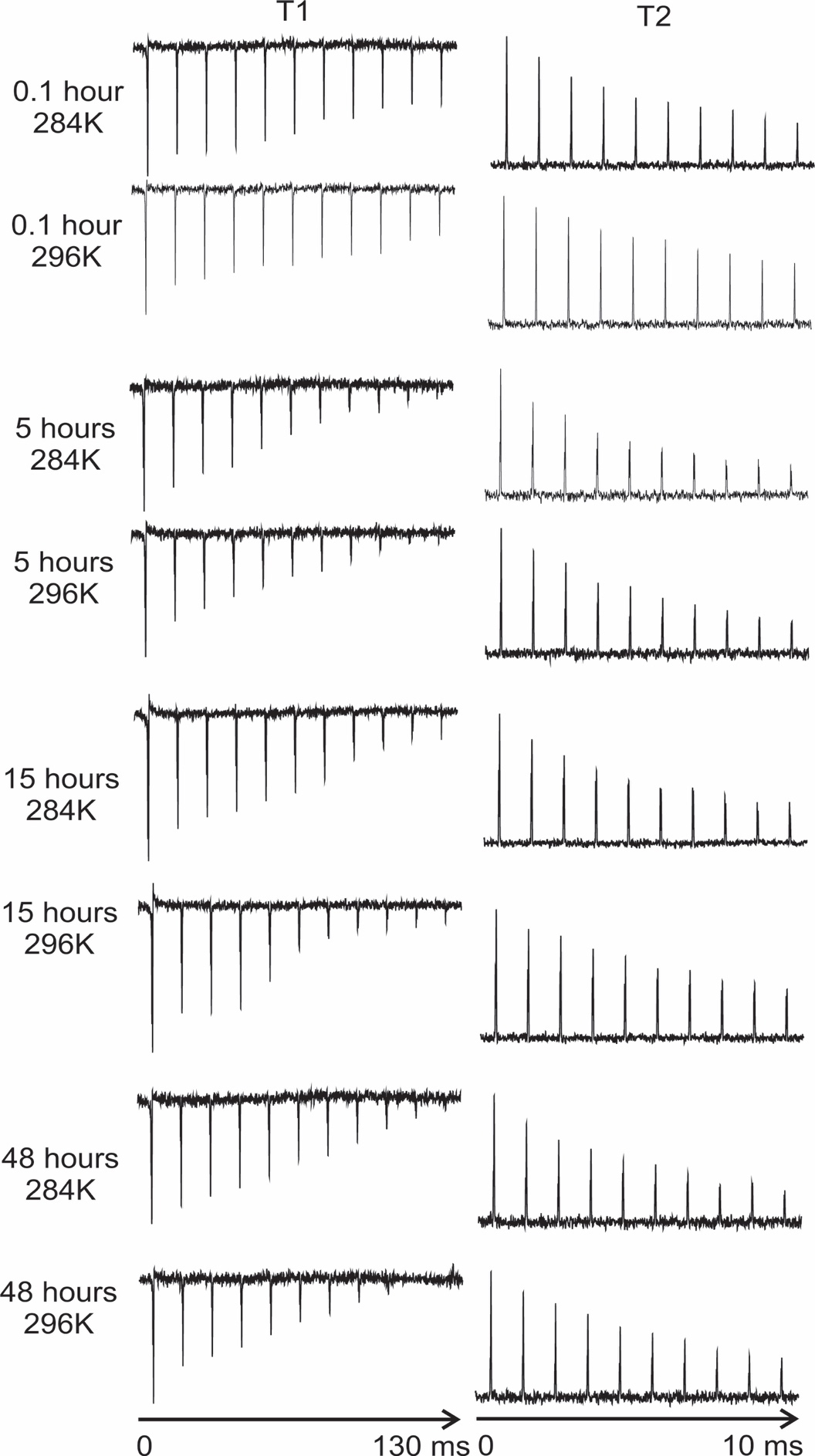


**Figure S4** Representative ^31^P relaxation spectra acquired on rSPMs-associated Aβ_1-40_.


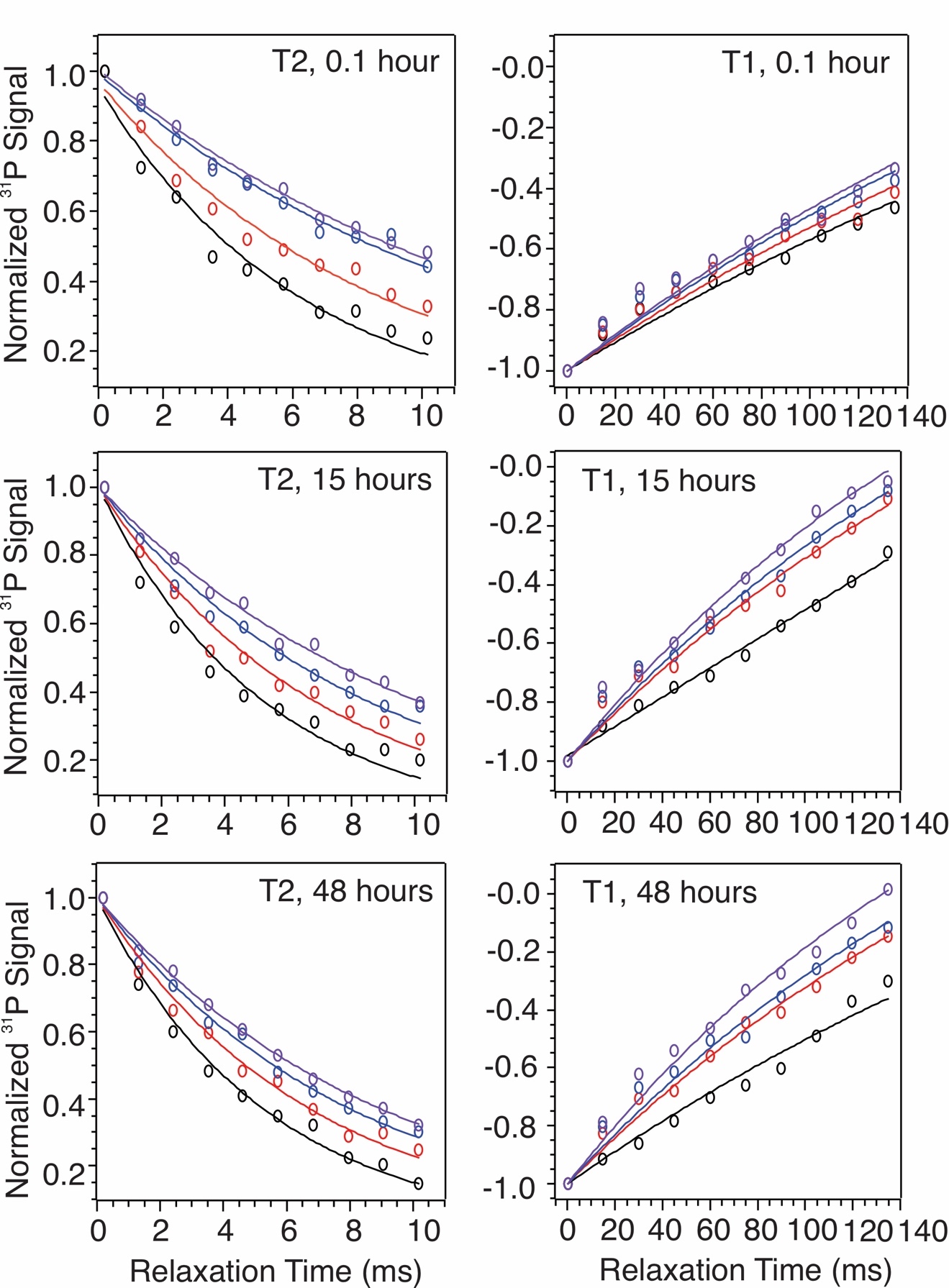


**Figure S5** Additional ^31^P relaxation curves with 0, 15 and 48-hour incubation times. Color-coding, black, 278K, red, 284K, blue, 290K and purple, 296K.


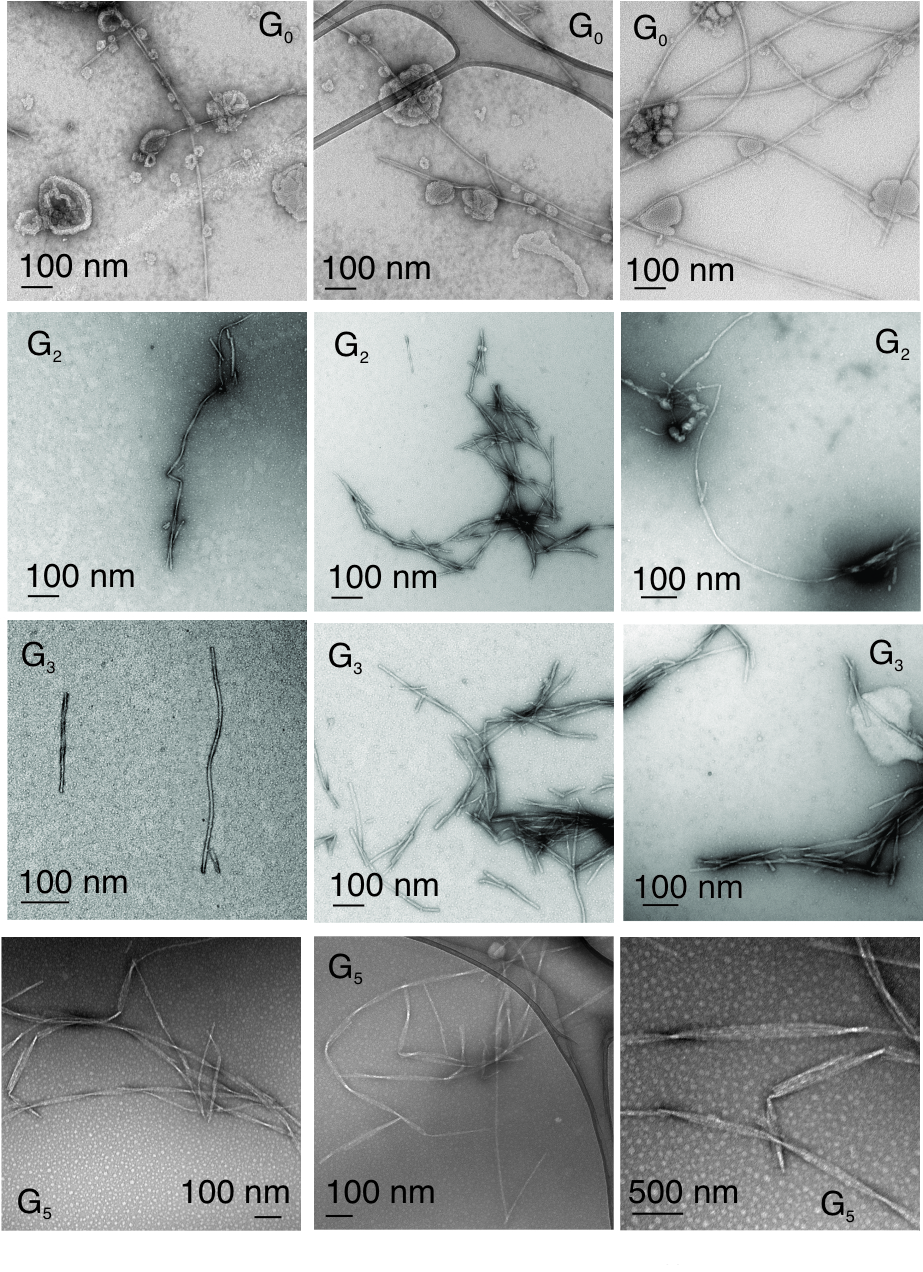


**Figure S6** Negatively stained TEM images for the parent G_0_, G_2_, G_3_ and G_5_ rSPMs-Aβ_1-40_ fibrils.

**
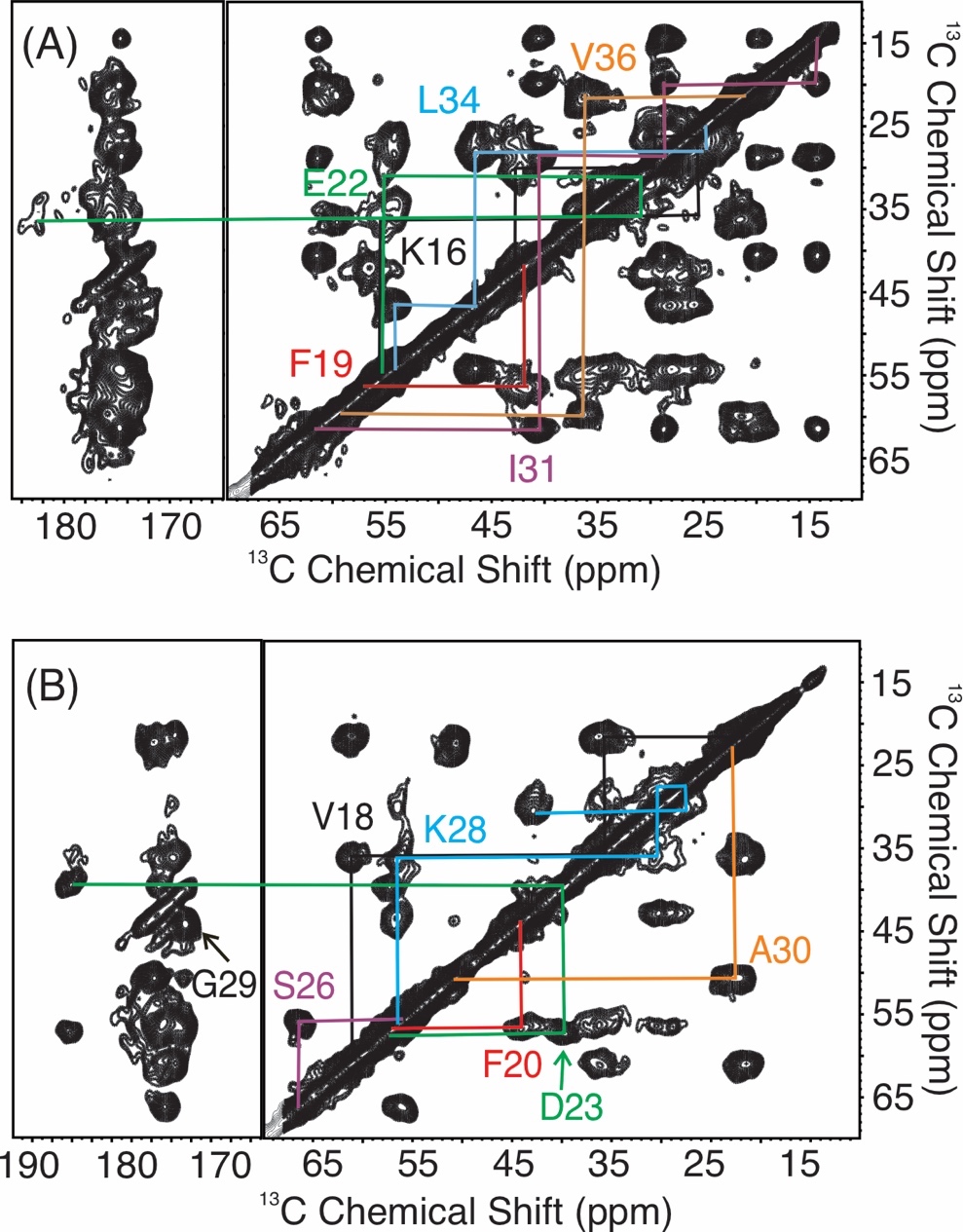
**

**Figure S7** Additional representative 2D ^13^C-^13^C spin-diffusion ssNMR spectra with 20 ms mixing period (uniformly ^13^C-labeled at (A) A2, S8, K16, F19, E22, I31, L34, V36, G37; and (B) E11, V18, F20, D23, S26, K28, G29, A30). Intra-residue cross peaks are highlighted using colored lines.

**
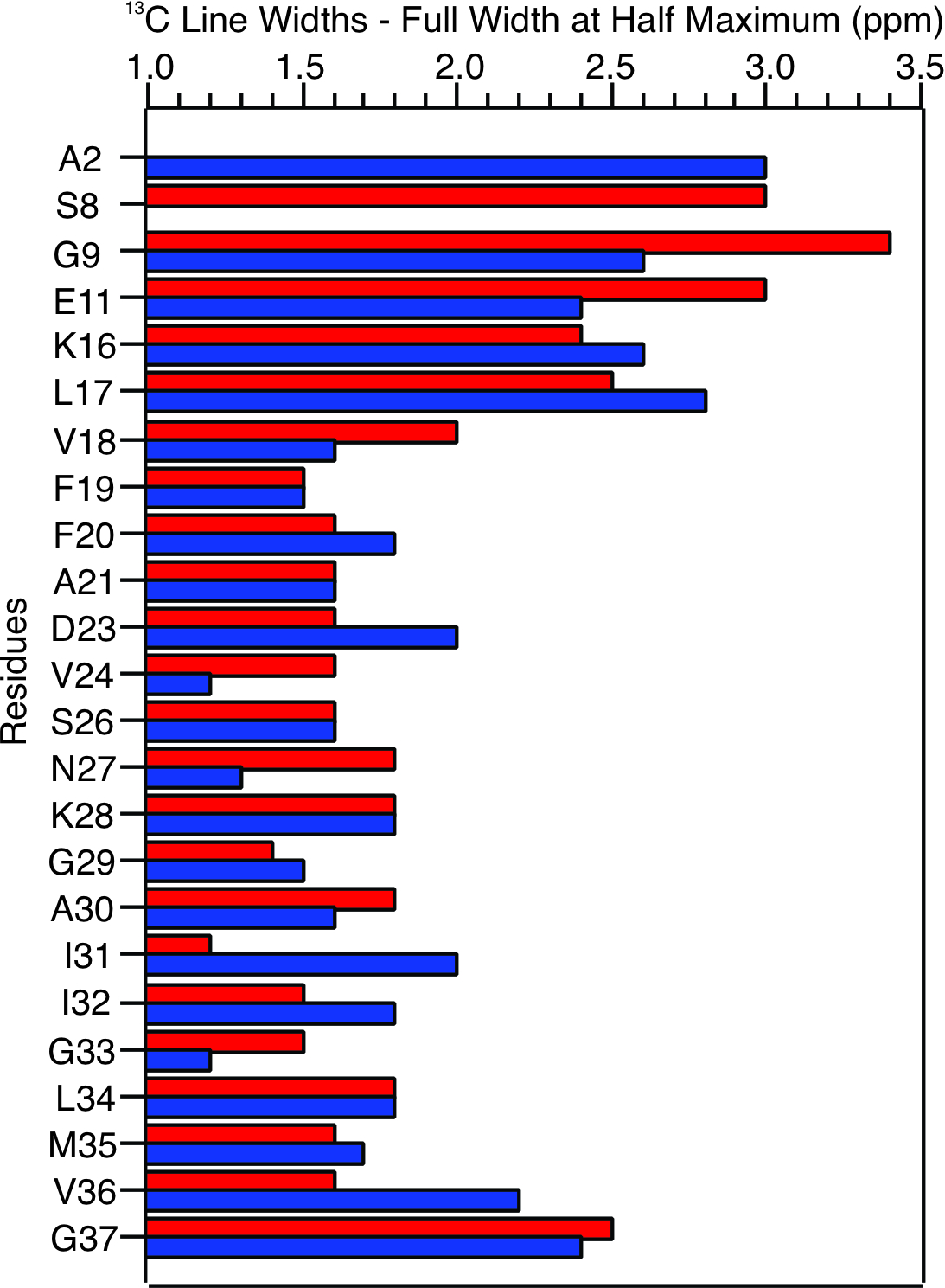
**

**Figure S8** Plots of the residue-specific ^13^C line widths (full width at the half maximum, FWHM) for C’ (red bars, determined based on the C’/Cα intra-residue cross peaks) and Cα (blue bars, determined based on the Cα/Cβ intra-residue cross peaks).

**
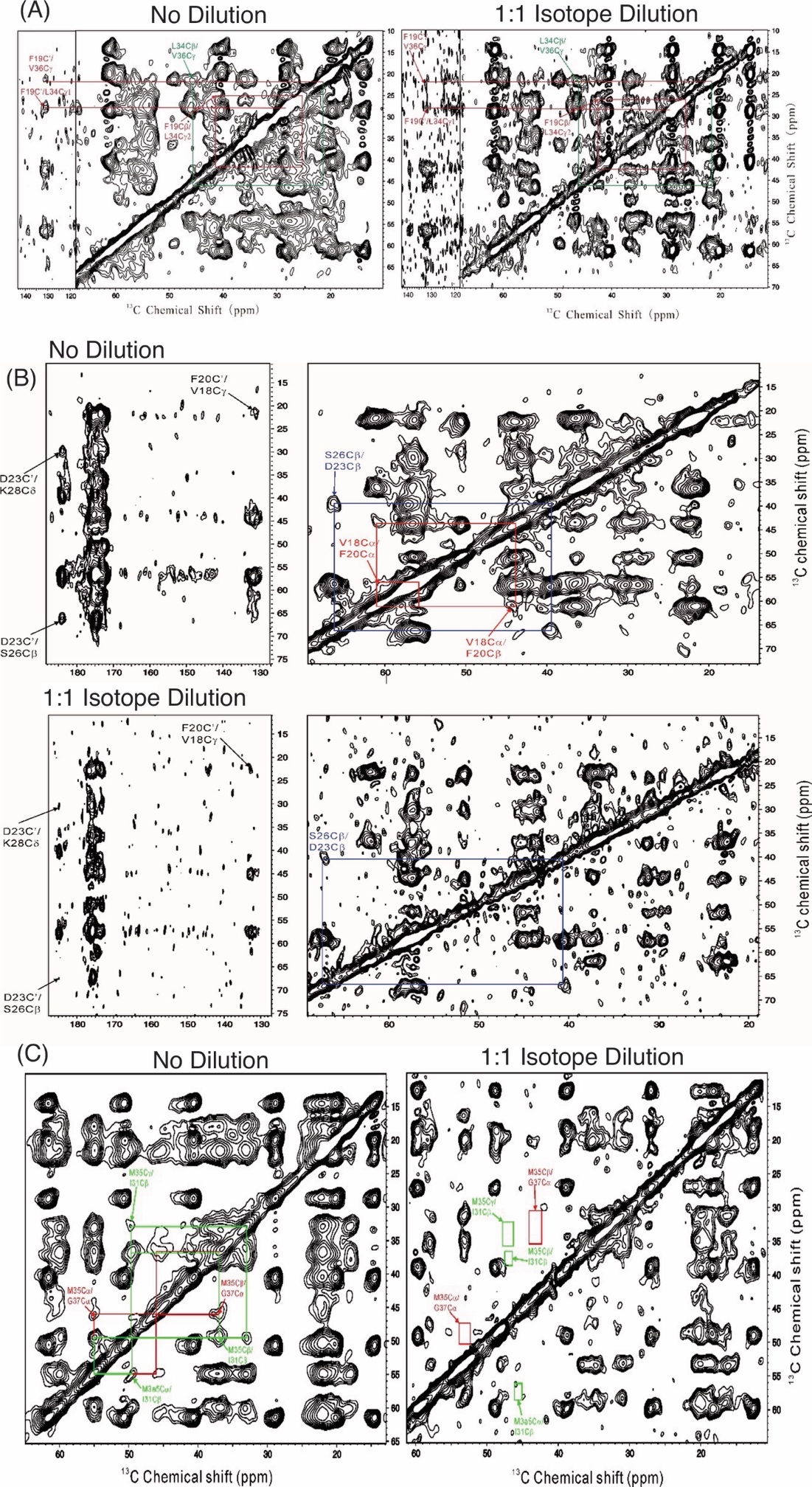
**

**Figure S9** Sample 2D ^13^C-^13^C spin diffusion spectra with 500 ms for rSPMs-Aβ_1-40_ fibrils with uniform ^13^C labeling at (A) A2, S8, K16, F19, E22, I31, L34, V36, G37, (B) E11, V18, F20, D23, S26, K28, G29, A30, and (C) A30, I31, M35, V36 and G37.


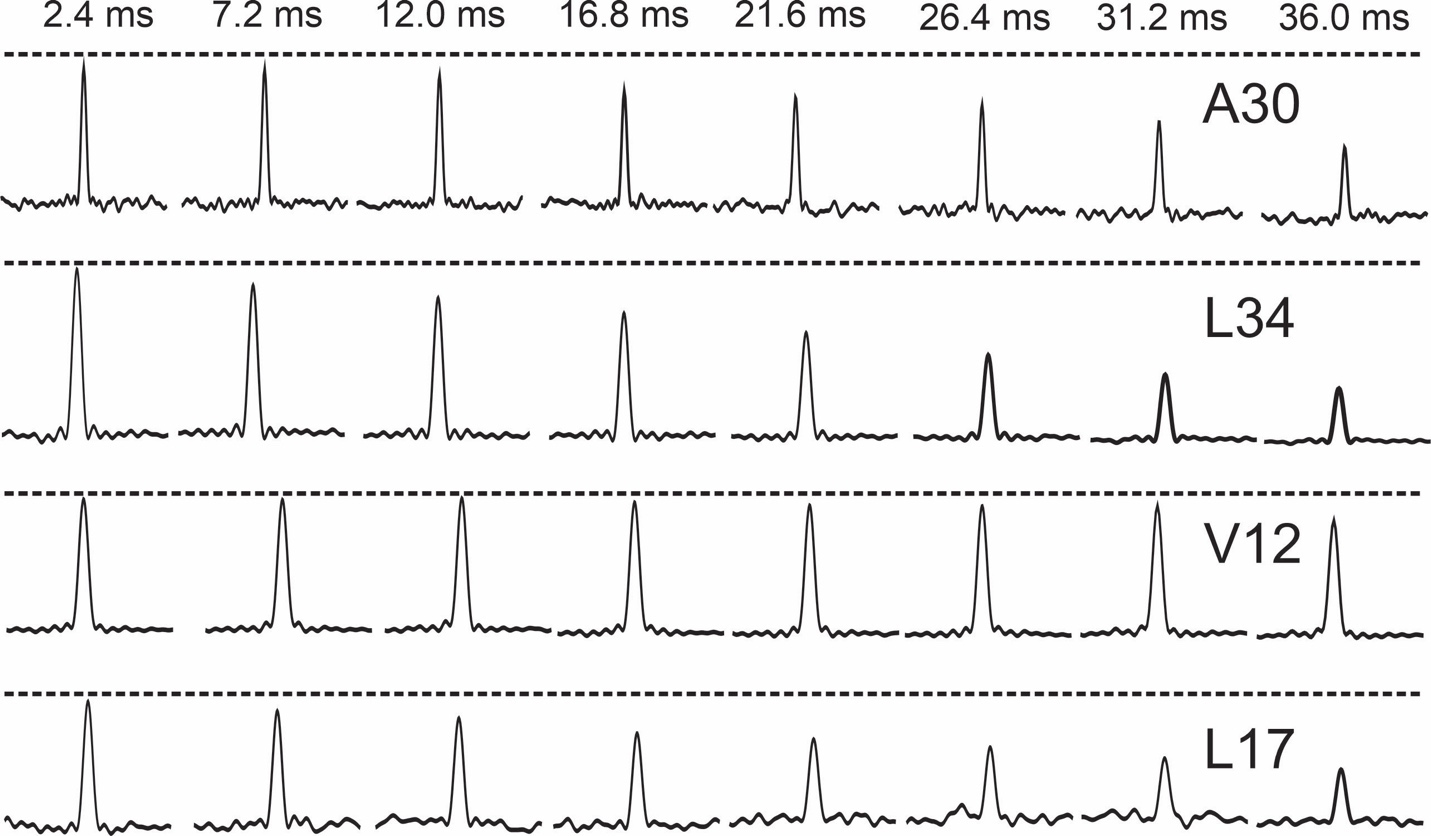


**Figure S10** Representative ^13^C-PITHIRDs spectra for A30-Cβ, L34-C’, V12-C’ and L17-C’ for rSPMs-Aβ_1-40_ fibrils. All spectra were collected with pulsed spin locking acquisition algorithm so that the transmitter was moved to the targeting resonance peak. Therefore, no chemical shift information was obtained.


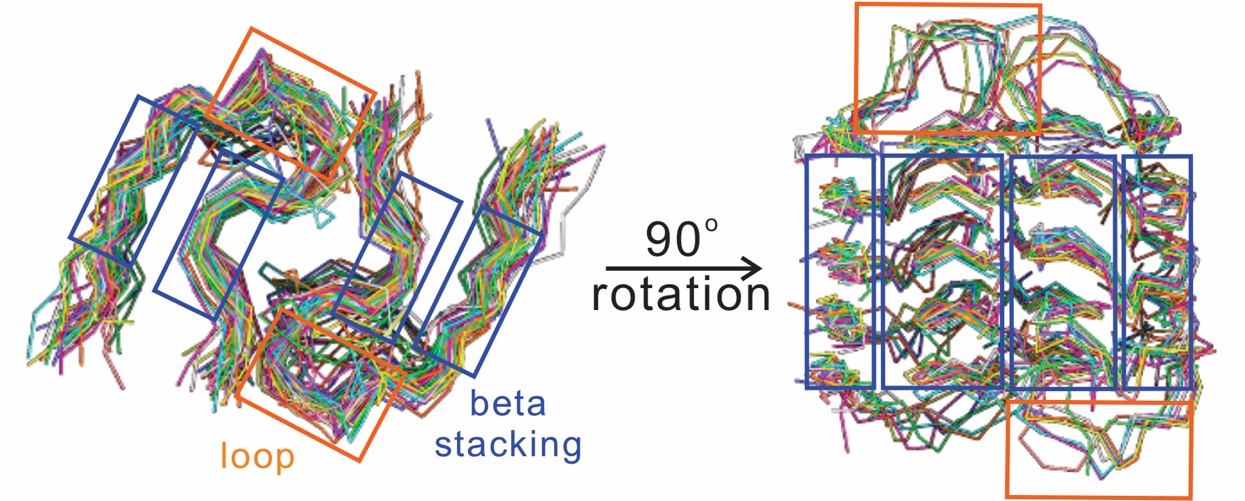


**Figure S11** Overlapping of eight lowest-energy rSPMs-Aβ_1-40_ structural models (backbone only). The blue and orange rectangles highlight the rigid beta-sheet stacking and more disordered loop segments, respectively. The RMSDs for the backbone Cα of residues 16-22, 30-36 and 23-29 are 1.499, 1.052 and 2.499 Å, respectively.

**Figure S12** Cell viability of N2a cells with the addition of Aβ_40_ fibrils grown from different membrane environments (including model phospholipid bilayers and isolated synaptic plasma membranes): MM1, PC/PG with molar ratio 3:1; MM4, PC/PG/cholesterol with molar ratio 3:1:1.5; MM5, PC/PG/cholesterol/sphingomyelin/gangliosides with molar ratio 3:1:1.5:1.5:0.3; BM1-BM3, the rSPMs from 3-month, 12-month and 18-month rats, respectively.

***S.I. Tables***

**Table S1** Table 1 Residue-specific ^13^C Chemical Shift Assignment of rSPMs-Aβ_1-40_ fibril.

|  | C’ (ppm) | Cα (ppm) | Cβ (ppm) | Cγ (ppm) | Cδ (ppm) | Cε (ppm) |
| --- | --- | --- | --- | --- | --- | --- |
| A2 | n.a. | 55.7 | 23.9 |  |  |  |
| S8 | 175.1 | 57.7 | 64.9 |  |  |  |
| G9 | 170.7 | 44.7 |  |  |  |  |
| E11 | 175.1 | 54.8 | 33.7 | 38.6 | 182.9 |  |
| K16 | 175.8 | 55.7 | 36.8 | 26.8 | 30.8 | 42.9 |
| L17 | 174.0 | 53.6 | 44.2 | 29.3  27.3 | 24.6 |  |
| V18 | 173.6 | 61.1 | 36.4 | 21.7 |  |  |
| F19 | 173.9 | 56.4 | 41.8 |  |  |  |
| F20 | 172.6 | 57.0 | 43.7 |  |  |  |
| A21 | 174.5 | 49.3 | 22.6 |  |  |  |
| E22 | 175.7 | 54.3 | 34.2 | 37.2 | 182.2 |  |
| D23 | 175.2 | 57.3 | 39.6 | 184.2 |  |  |
| V24 | 175.8 | 59.5 | 33.2 | 20.5 |  |  |
| S26 | 174.6 | 56.4 | 66.4 | 22.1 |  |  |
| N27 | 173.3 | 52.3 | 40.5 | 175.7 |  |  |
| K28 | 174.0 | 56.5 | 36.0 | 27.7 | 30.2 | 42.7 |
| G29 | 172.6 | 44.0 |  |  |  |  |
| A30 | 175.8 | 50.7 | 22.1 |  |  |  |
| I31 | 174.8 | 61.5 | 40.8 | 28.9  19.9 | 14.3 |  |
| I32 | 175.7 | 57.3 | 41.7 | 26.5  16.8 | 13.7 |  |
| G33 | 171.5 | 48.6 |  |  |  |  |
| L34 | 174.9 | 54.2 | 46.7 | 28.3  27.5 | 26.0 |  |
| M35 | 173.3 | 53.8 | 36.8 | 31.8 | 16.8 |  |
| V36 | 175.8 | 59.8 | 36.4 | 21.5 |  |  |
| G37 | 173.9 | 50.0 |  |  |  |  |
| ^1^Chemical shift or linewidths cannot be determined due to spectral overlapping; ^2^Full width at the half maximum (FWHM) in ppm unit. | | | | | | |

**Table S2** Isotope labeling schemes and the utilized ssNMR spectroscopy

| Labeling Schemes | ssNMR experiments |
| --- | --- |
| ^13^C-U-G9, L17, A21, V24, N27, I32, G33, M35 | 2D-PDSD (20ms and 500ms, non-diluted and 1:1 isotope-diluted) |
| ^13^C-U-A2, S8, K16, F19, E22, I31, L34, V36, G37 | 2D-PDSD (20ms and 500ms, non-diluted and 1:1 isotope-diluted) |
| ^13^C-U-E11, V18, F20, D23, S26, K28, G29, A30 | 2D-PDSD (20ms and 500ms, non-diluted and 1:1 isotope-diluted) |
| ^13^C-U-A30, I31, M35, V36, G37 | 2D-PDSD (500ms, non-diluted and 1:1 isotope-diluted) |
| ^13^C-U-I31, M35, G38, V39 | 2D-PDSD (20ms, non-diluted) |
| ^13^C-L17(C’), A21(Cβ), G33(Cα) | ^13^C-PITHIRDs-CT, mature fibril |
| ^13^C-V12(C’), A30(Cβ), G37(Cα) | ^13^C-PITHIRDs-CT, mature fibril |
| ^13^C-V18(C’), A2(Cβ), G29(Cα) | ^13^C-PITHIRDs-CT, mature fibril |
| ^13^C-L34(C’) | ^13^C-PITHIRDs-CT, mature fibril, short incubation, ^13^C-^31^P REDOR |
| ^13^C-V24(C’) | ^13^C-PITHIRDs-CT |
| ^13^C-L19(C’), A21(Cβ), G29(Cα) | ^13^C-PITHIRDs-CT, short incubation, ^13^C-^31^P REDOR |
| ^13^C-V36(C’), A30(Cβ), G25(Cα) | ^13^C-PITHIRDs-CT, short incubation, ^13^C-^31^P REDOR |

**Table S3** Key inter-residue cross peaks and the isotope dilution effect.

| Site 1 | Site 2 | Percentage of Volume Reduction due to Isotope Dilution |
| --- | --- | --- |
| K16-Cε | L34-Cγ | 25.0 ± 4.9 |
| K16-Cε | L34-Cβ | 30.1 ± 6.5 |
| K16-Cε | L34-Cδ1 | 22.9 ± 4.7 |
| F19-Cζ | L34-Cγ | 26.3 ± 7.7 |
| F19-Cβ | L34-Cγ | 31.3 ± 6.2 |
| A21-Cβ | I32-Cβ | 40.9 ± 8.2 |
| A21-Cβ | I32-Cγ2 | 56.6 ± 5.9 |
| A21-Cβ | I32-Cδ1 | 25.5 ± 7.1 |
| I32-Cδ1 | V24-Cα | 51.9 ± 7.7 |
| I32-Cδ1 | V24-Cγ1 | 55.3 ± 6.6 |
| I32-Cγ1 | V24-Cα | 68.2 ± 6.1 |
| I32-Cγ2 | V24-Cα | 66.3 ± 5.4 |
| I32-Cβ | V24-Cα | 59.9 ± 7.2 |
| I32-Cα | V24-Cγ1 | 70.5 ± 5.9 |
| I32-Cβ | V24-Cγ1 | 52.2 ± 6.6 |
| G33-Cα | V24-Cα | 64.2 ± 5.3 |
| D23-Cγ | S26-Cβ | 83.6 ± 4.4 |
| D23-Cβ | S26-Cβ | 80.9 ± 4.5 |
| A30-Cα | M35-Cδ | 9.0 ± 7.2 |
| I31-Cβ | M35-Cδ | 15.0 ± 4.2 |
| I31-Cγ1 | M35-Cδ | 31.2 ± 6.5 |
| I31-Cδ1 | M35-Cδ | 11.1 ± 6.0 |
| A30-Cα | M35-Cβ | 14.2 ± 7.3 |
| A30-Cα | V36-Cα | 22.9 ± 7.0 |
